# An engineered photoswitchable mammalian pyruvate kinase

**DOI:** 10.1111/febs.14175

**Published:** 2017-08-16

**Authors:** Stefanie Gehrig, Jamie A. Macpherson, Paul C. Driscoll, Alastair Symon, Stephen R. Martin, James I. MacRae, Jens Kleinjung, Franca Fraternali, Dimitrios Anastasiou

**Affiliations:** ^1^ Cancer Metabolism Laboratory The Francis Crick Institute London UK; ^2^ Metabolomics Science Technology Platform The Francis Crick Institute London UK; ^3^ Instrument Prototyping Science Technology Platform The Francis Crick Institute London UK; ^4^ Structural Biology Science Technology Platform The Francis Crick Institute London UK; ^5^ Computational Biology The Francis Crick Institute London UK; ^6^ Randall Division of Cell and Molecular Biophysics King's College London UK

**Keywords:** LOV2, metabolism, molecular dynamics, optogenetics, PKM2

## Abstract

Changes in allosteric regulation of glycolytic enzymes have been linked to metabolic reprogramming involved in cancer. Remarkably, allosteric mechanisms control enzyme function at significantly shorter time‐scales compared to the long‐term effects of metabolic reprogramming on cell proliferation. It remains unclear if and how the speed and reversibility afforded by rapid allosteric control of metabolic enzymes is important for cell proliferation. Tools that allow specific, dynamic modulation of enzymatic activities in mammalian cells would help address this question. Towards this goal, we have used molecular dynamics simulations to guide the design of mPKM2 internal light/oxygen/voltage‐sensitive domain 2 (LOV2) fusion at position D24 (PiL[D24]), an engineered pyruvate kinase M2 (PKM2) variant that harbours an insertion of the light‐sensing LOV2 domain from *Avena Sativa* within a region implicated in allosteric regulation by fructose 1,6‐bisphosphate (FBP). The LOV2 photoreaction is preserved in the PiL[D24] chimera and causes secondary structure changes that are associated with a 30% decrease in the *K*_m_ of the enzyme for phosphoenolpyruvate resulting in increased pyruvate kinase activity after light exposure. Importantly, this change in activity is reversible upon light withdrawal. Expression of PiL[D24] in cells leads to light‐induced increase in labelling of pyruvate from glucose. PiL[D24] therefore could provide a means to modulate cellular glucose metabolism in a remote manner and paves the way for studying the importance of rapid allosteric phenomena in the regulation of metabolism and enzyme control.

**Abbreviations**
ADP: adenosine diphosphate
ATP: adenosine triphosphate
CD: circular dichroism
FBP: fructose 1,6‐bisphosphate
FMN: flavin mononucleotide
GC‐MS: gas chromatography‐mass spectrometry
(h/m)PKM2: (human/mouse) pyruvate kinase M2 isoform
HLH: helix‐loop‐helix
LDH: lactate dehydrogenase
LED: light‐emitting diode
LOV2: light/oxygen/voltage‐sensitive domain 2
MD: molecular dynamics
MRW: mean residue weight
NAD(H): nicotinamide adenine dinucleotide (reduced)
NMR: nuclear magnetic resonance
PAS: Per‐Arnt‐Sim
PCA: principal component analysis
PCR: polymerase chain reaction
PDB: Protein Data Bank
PEP: phosphoenolpyruvate
PiL[D24]: mPKM2 internal LOV2 fusion at position D24
PKM1: pyruvate kinase M1 isoform
SAICAR: succino‐5‐aminoimidazole‐4‐carboxamide ribonucleotide
UV: ultraviolet

## Introduction

Allostery is a ubiquitous regulatory mechanism used to control a wide spectrum of molecular processes 1, 2, 3, 4. The functional response of proteins to allosteric ligand binding typically occurs within the nanosecond to millisecond time range 5 and this speed confers the necessary flexibility for cells to adapt to various stimuli and changes in their environment. Mutagenesis of protein residues involved in allostery can be used to probe the function of specific allosteric mechanisms in cellular physiology. However, this approach has limitations 6, 7 as the effects of allosteric mutants are not readily reversible at allosteric time‐scales and can be masked by compensatory mechanisms that overcome the functional consequences of the mutant 8. Tools that allow reversible control of allostery in mammalian cells are not widely available, so it remains unclear if the rapid dynamics of allosteric control influence long‐term cellular physiology.

Alterations in allosteric properties of proteins have been linked to disease 9, 10. This is well exemplified in the field of cancer metabolism where expression of enzymes that have different allosteric properties to those of their counterparts expressed in healthy cells underlie some of the metabolic features of tumours. Selective isoform expression and the dependency of tumours on specific metabolic pathways have raised hopes that, despite the ubiquity of metabolic processes, metabolism in cancer cells can be specifically targeted for therapy 10. Indeed, allosteric pockets can provide advantages over orthosteric molecules for therapy 11, 12. Understanding how allostery contributes to altered metabolism in disease is, therefore, likely to yield useful insights towards rational therapeutic strategies.

Glucose metabolism is particularly important for many tumours and among several glycolytic enzymes that have been implicated in cancer PKM2 has a pivotal role in determining the fate of glucose carbons to support tumorigenesis 13, 14, 15, 16, 17. PKs catalyse the conversion of phosphoenolpyruvate and adenosine diphosphate (ADP) to pyruvate and adenosine triphosphate (ATP). Among the four known PK isoforms found in mammals, PKM2 is expressed in many cancer cells as well as various healthy cells 18, 19. PKM2 is encoded by the *Pkm* gene along with the closely related splice variant pyruvate kinase M1 isoform (PKM1). Unlike PKM1, which is constitutively active, PKM2 is allosterically activated by a variety of intracellular ligands, including the glycolytic intermediate fructose 1,6‐bisphosphate (FBP) 20, the amino acid L‐serine 17 and the nucleotide synthesis intermediate succino‐5‐aminoimidazole‐4‐carboxamide ribonucleotide (SAICAR) 21.

In tumour cells, various conditions such as aberrant growth factor signalling and oxidative stress inhibit PKM2, which results in the diversion of glucose carbons into anabolic and redox regulating pathways that are essential for cell growth and survival 16, 17, 22, 23, 24. Intriguingly, despite high expression in tumours, genetic abrogation of PKM2 does not prevent tumour growth in mice 25, 26. Re‐expression of PKM1 can occur in nonproliferating tumour cells 25 and several parenchymal cells 19. However, proliferating PKM2‐null tumour cells have no detectable PK expression, which likely reflects an adaptation that suppresses expression of PKM1 in these tumours 25. Consistent with a negative role of high PK activity in tumour growth, both exogenous expression of PKM1 or pharmacological activators that overcome endogenous PKM2‐inhibiting mechanisms impede tumour growth by increasing cellular PK activity, effectively rendering endogenous PKM2 into a PKM1‐like enzyme 13. These observations indicate that the ability to down‐regulate cellular PK activity by expression of the PKM2 isoform confers properties to the metabolic network that are conducive to tumorigenesis. However, the fact that tumours grow in the absence of detectable PK indicates that lowering PK expression, or expressing an isoform with constitutively low activity should suffice to support tumour growth. So, the advantages of expressing an isoform that can be dynamically regulated through allostery are unclear.

Engineering a pyruvate kinase that can be controlled remotely and reversibly would provide a means to explore the role of rapid allosteric phenomena in cellular metabolism. Towards this goal, here we present the design and characterisation of a photoswitchable PKM2 variant, mPKM2 internal light/oxygen/voltage‐sensitive domain 2 (LOV2) fusion at position D24 (PiL[D24]), that harbours an insertion of the light‐sensitive *Avena sativa* (oat) LOV2 domain. PiL[D24] activity is reversibly controlled by light and, when expressed in cells, it allows remote modulation of cellular pyruvate kinase activity.

## Results

### Molecular dynamics simulations identify conformational changes in PKM2 associated with FBP binding

**Figure 1:**
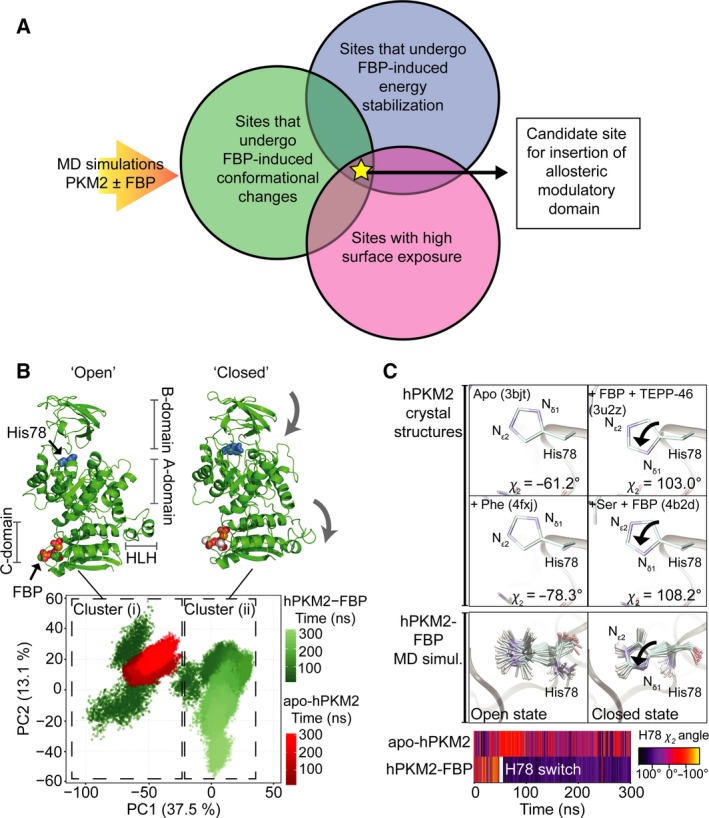
MD reveals conformational changes on PKM2 upon FBP binding. (A) Summary of criteria that led to the identification of a candidate site used for inserting a heterologous allosteric domain to generate a photoswitchable pyruvate kinase. MD simulations of PKM2 with and without the allosteric activator FBP were used to identify residues that undergo FBP‐induced conformational changes (green circle), show energetic stabilisation indicative of allosteric communication with the FBP‐binding pocket (blue circle) and were surface‐exposed (pink circle). A site that fulfilled all three criteria was used to insert a light‐sensory domain. (B) MD simulations of apo‐hPKM2 (red) and hPKM2‐FBP (green) were projected onto the first two principal components (PCs) determined from the PCA of the hPKM2‐FBP trajectory. The time evolutions of both the apo‐ and FBP‐bound hPKM2 MD trajectories, from 0 to 300 ns, are represented as colour gradients. A *k*‐means clustering of the PCA plot identified two distinct Clusters (i) and (ii) (dashed boxes) that explained 100% of the point variability of the data set. Single most dominant conformations of hPKM2‐FBP were extracted from each of these two clusters and are shown in cartoon representations; FBP and the catalytic histidine (His78, blue) are shown as spacefill representations. Comparison of the structures extracted from Cluster (i) and Cluster (ii) revealed two dominant conformational motions (indicated with the grey arrows) as the protein transitioned from Cluster (i) to Cluster (ii): closure of the B‐domain and flipping downward of the HLH, which led us to assign Clusters (i) and (ii) as ‘open’ and ‘closed’ conformation respectively. (C) A superimposition of hPKM2 active site residues revealed that His78 adopts an altered side‐chain conformation in the crystal structure when the protein is bound to activators fructose 1,6‐bisphosphate (FBP) and TEPP‐46 (PDB ID: 3u2z) and when bound to L‐serine and FBP (PDB ID: 4b2d), compared to crystal structures of apo‐hPKM2 (PDB ID: 3bjt) and hPKM2 in complex with the allosteric inhibitor L‐phenylalanine (PDB ID: 4fxj). In the structures bound to the activators, the N_δ1_ nitrogen group is positioned pointing towards the substrate‐binding pocket, and is flipped away from the substrate‐binding pocket in the apo‐ and phenylalanine‐bound structures. Consistent with this change following allosteric ligand binding, the conformation of His78 can provide a read‐out for whether PKM2 is in the catalytically active or inactive state. Below, 18 evenly spaced snap‐shots of the conformation of His78 are shown for a MD simulation of hPKM2‐FBP in the ‘open’ and following the transition to the ‘closed’ states. In the ‘open’ state [Cluster (i) in Fig. 1B], the side chain of His78 is flexible and switches into a conformation seen in that of the active crystal structures following the transition of the simulation into cluster ii [Cluster (ii) in Fig. 1B]. The time evolution of the χ_2_ dihedral angle of His78 along MD simulations of apo‐hPKM2 and hPKM2‐FBP, colour‐coded according to the scale on the right. The His78 switch occurs after ~ 50 ns (hPKM2‐FBP), while apo‐hPKM2 remains flexible for the duration of the simulation.

Various intracellular mechanisms in cancer cells maintain PKM2 in a low‐activity monomeric state 16, 17, 22, 23, 24 and forced stabilisation of tetrameric PKM2 increases its enzymatic activity thereby impeding cell proliferation 13, 15. Although modulation of oligomerisation would be a potential way to exogenously control PKM2 activity, this could be particularly challenging, as it would necessitate the design of an engineered protein that can tetramerise reversibly. Allosteric ligands often exert reversible effects on enzymatic activity by inducing conformational changes to the protein, so we reasoned that mimicking ligand‐induced conformational changes could provide a means to modulate activity. We therefore set out to identify conformational changes linked to binding of the major PKM2 allosteric ligand FBP and determine a suitable method to modulate these (summarised in Fig. 1A).

To investigate the mechanical response of PKM2 upon binding of FBP, we performed molecular dynamics (MD) simulations of the human PKM2 (hPKM2) monomer in the absence (apo‐hPKM2) or presence of FBP (hPKM2‐FBP). For this analysis, we selected the human orthologue due to availability of crystal structures needed to seed MD simulations. A principal component analysis (PCA) of the simulations revealed that hPKM2‐FBP sampled two discrete conformational states [Fig. 1B, Clusters (i) and (ii), as identified by a *k*‐means clustering]. Transition from Cluster (i) to Cluster (ii) was dominated by the closure of the B‐domain over the substrate‐binding pocket and the ‘flipping’ of the N‐terminal helix‐loop‐helix (HLH) into an alternate, stable conformation. This transition into what we here define as the ‘closed’ conformation [seen in Cluster (ii)] was accompanied by the catalytic His78 20 adopting a side‐chain conformation that is similar to that found in crystal structures of PKM2 bound to activators (Fig. 1C). Conversely, simulations of apo‐hPKM2 only sampled a restricted conformational space within Cluster (i) with the B‐domain remaining in the ‘open’ conformation. Taken together these observations suggest that the B‐domain and HLH motions occur in an FBP‐dependent manner and that the closed conformation may represent the active state of the enzyme.

**Figure 2:**
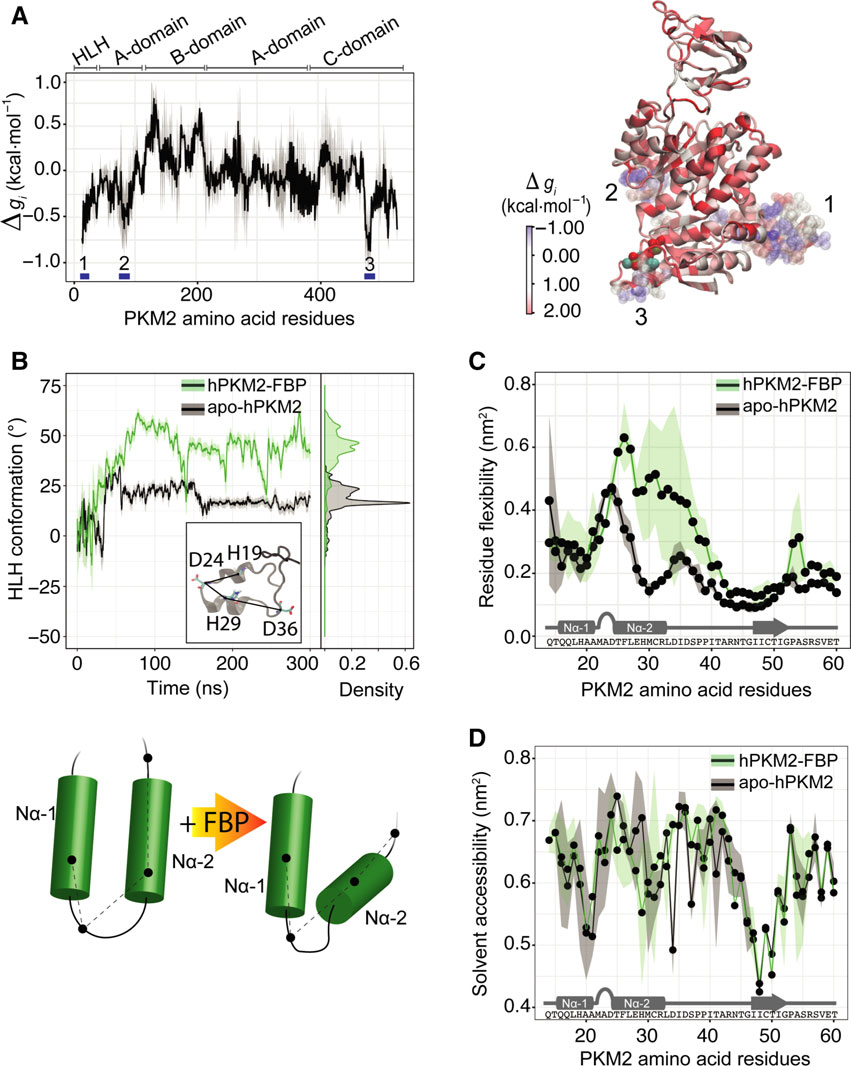
Identification of D24 as a candidate site for insertion of the LOV2 domain. (A) The per‐residue free energy difference ∆*g*_*i*_ between the apo‐ and FBP‐bound MD simulations were calculated according to the method presented in 27, giving the difference in the amount of configurational work exerted on each residue *i* due to allosteric ligand binding. The numbers and blue bars indicate the HLH 1, FBP‐binding pocket 3 and a third site encompassing residues 86–97 2 that are energetically stabilised by FBP binding (negative on the ∆*g*_*i*_ scale). Conversely, residues in the B‐domain yield a significant increase of configurational work (positive on the ∆*g*_*i*_ scale) as a result of allosteric communication between sites. The right panel shows a cartoon representation of these data coloured according to the configurational work exerted per residue in the corresponding part of the protein (negative values in blue showing energetic stabilisation; and positive values in red showing increase in local configurational work). (B) A planar dihedral angle was defined between the Cα atoms of His19, Asp24, His29 and Asp36 (see inset) to quantify, in a time‐resolved manner, conformational changes of the N‐terminal HLH in the MD simulations of apo‐hPKM2 (black) and hPKM2‐FBP (green). Running averages and standard deviations of the dihedral angle over the trajectories were calculated with sliding windows of 1 ns and are shown as solid and transparent lines respectively. A density histogram showing the distribution of the dihedral angle is shown. The lower panel shows a cartoon representing the observed displacement of the Nα‐2 helix by a hinge‐like motion. (C) The root‐mean‐square deviations of the Cα atoms were measured from the apo‐hPKM2 and hPKM2‐FBP MD simulations averaged over three replicates for each state respectively. The points indicate the averaged root‐mean‐square deviation over the three replicates and the shading shows the standard deviation of the mean. The second helix (Nα2) in the HLH motif is more flexible upon FBP binding, permitting the observed ‘flipping’ down in the presence of the allosteric activator. (D) Plotting of the time‐averaged solvent accessibility calculated from the MD simulations of apo‐hPKM2 and hPKM2‐FBP shown for the first 60‐amino‐acid residues, which reveals that residues in the interhelical loop are highly solvent exposed (including residue D24). The points indicate the averaged solvent accessibility over the three replicates and the shading shows the standard deviation of the mean.

We also used an orthogonal method to independently identify residues involved in the FBP‐induced allosteric response of PKM2. Local energetic changes that occur at distal sites within a protein upon ligand binding underlie allosteric communication between such sites 27, 28, 29. To identify residues responsible for FBP‐induced allosteric activation, we estimated the per‐residue free energy difference between the FBP‐bound and apo‐hPKM2 MD simulations 27 (Fig. 2A). In addition to residues in the FBP‐binding pocket (site #3 in Fig. 2A, ∆*g*_(FBP)_ = −0.43 kcal·mol^−1^), ligand binding was predicted to contribute to the energetic stabilisation of residues 14–40 in the HLH (site #1, ∆*g*_(14–40)_ = −0.32 kcal·mol^−1^) and residues 86–97 (site #2, ∆*g*_(86–97)_ = −0.21 kcal·mol^−1^) adjacent to the catalytic pocket. Energetic stabilisation of these residues supports the idea that their dynamics are allosterically coupled to FBP binding. Intriguingly, small molecule activators bind at a region proximal to the HLH and activate PKM2 13, further corroborating the idea that the HLH is involved in allosteric regulation of the enzyme. Combined, the analyses of the MD simulations revealed that FBP binding is associated with conformational changes in the HLH that likely mediate the allosteric effects of FBP to the catalytic pocket.

We considered how we could exploit the HLH conformational change to control PKM2 activity in a dynamic manner. This could be achieved by using a heterologous domain that is itself capable of undergoing reversible conformational changes following a remote stimulus. We hypothesised that, when inserted into the HLH, such a domain could undergo conformational changes that can be transmitted to the flanking PKM2. In plants and bacteria, specialised sensory domains undergo light‐induced conformational changes that allosterically regulate the activity of adjoining proteins to mediate organismal responses to light 30, 31. *Avena sativa* LOV2 (light‐oxygen‐voltage domain 2; henceforth referred to as LOV2 for simplicity) is a 16 kDa light‐sensory domain from phototropin 1 that undergoes flavin mononucleotide (FMN)‐dependent photocycles 32, 33. LOV2 comprises a Per‐Arnt‐Sim (PAS) core and a C‐terminal Jα‐helix. FMN renders LOV2 fluorescent but upon exposure to blue light, bound FMN forms a covalent bond with a cysteine residue in the PAS core, which results in loss of fluorescence and unfolding of the Jα‐helix, pushing the C terminus away from the PAS core 34, 35, 36, 37, 38, 39, 40. Importantly, the N and C termini of LOV2 are oriented towards the same direction making it suitable to insert into other proteins 41, 42, 43, and in the case of PKM2, into the HLH region. Furthermore, LOV2 is suitable for use in mammalian cells 44, which tolerate blue light well and do not need exogenous cofactors because they produce FMN from dietary vitamin B. Altogether, we concluded that LOV2 is a suitable domain to insert into PKM2 and potentially regulate its activity and for subsequent use in mammalian cells.

### The LOV2 photoswitch is preserved in PiL[D24]

**Figure 3:**
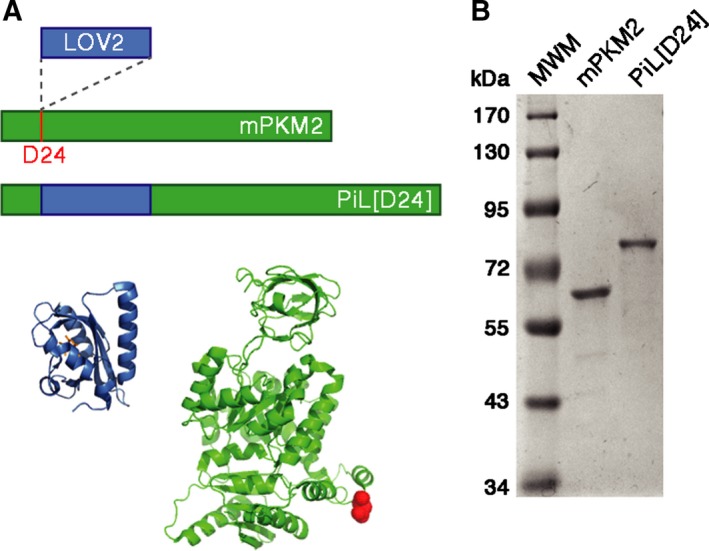
Construction of PiL[D24], a chimera of LOV2 and the LOV2 photosensory domain. (A) Schematic of the engineered PiL[D24] chimera showing the insertion of the light‐sensory LOV2 domain between residues Asp24 (D24) and Thr25 in mPKM2 to generate a light‐controlled PKM2 variant. Bottom panel left: Crystal structure of the *Avena Sativa* LOV2 domain (PDB ID: 2V0U) in blue with bound FMN in orange. Bottom panel right: Crystal structure of PKM2 (PDB ID: 3U2Z) in green. D24 is shown as red spheres and highlights the insertion site of LOV2 in the N‐terminal HLH of PKM2. (B) Coomassie‐stained SDS/PAGE of recombinant PKM2 (60 kDa) and PiL[D24] (75 kDa; 0.5 μg each) expressed in *Escherichia coli* and purified by affinity and SEC. MWM, molecular weight markers.

Closer inspection of the hPKM2‐FBP MD trajectories revealed that during the HLH ‘flipping’ motion, the N‐terminal helix (which we named Nα‐1) within the HLH is displaced by a hinge‐like mechanism (Fig. 2B). During this displacement, the Nα‐1 helix, as well as part of the interhelical loop (residues 14–24), remain relatively stable in their positioning, whereas the second helix (Nα‐2, residues 25–33) undergoes flexible dynamic motions over the course of the simulation with FBP (Fig. 2C). Furthermore, D24 is the most solvent‐exposed residue in the HLH (Fig. 2D) suggesting that an insertion of LOV2 at this position is likely to be tolerated without affecting the folding of the protein. We therefore cloned LOV2 (residues 404–540) 43, 45 between residues Asp24 and Thr25 in mouse PKM2 (mPKM2) to generate a chimeric protein henceforth referred to as PiL[D24] (Fig. 3A). To construct the chimera, we selected the mouse PKM2 orthologue with the outlook of using the resulting protein in cells, as it allows the concomitant knock‐down of endogenous PKM2 in human cells 14, 24. We expressed PiL[D24] in *Escherichia coli* as a His‐tagged protein, purified it by affinity chromatography (Fig. 3B) and set out to characterise its biophysical and enzymatic properties.

**Figure 4:**
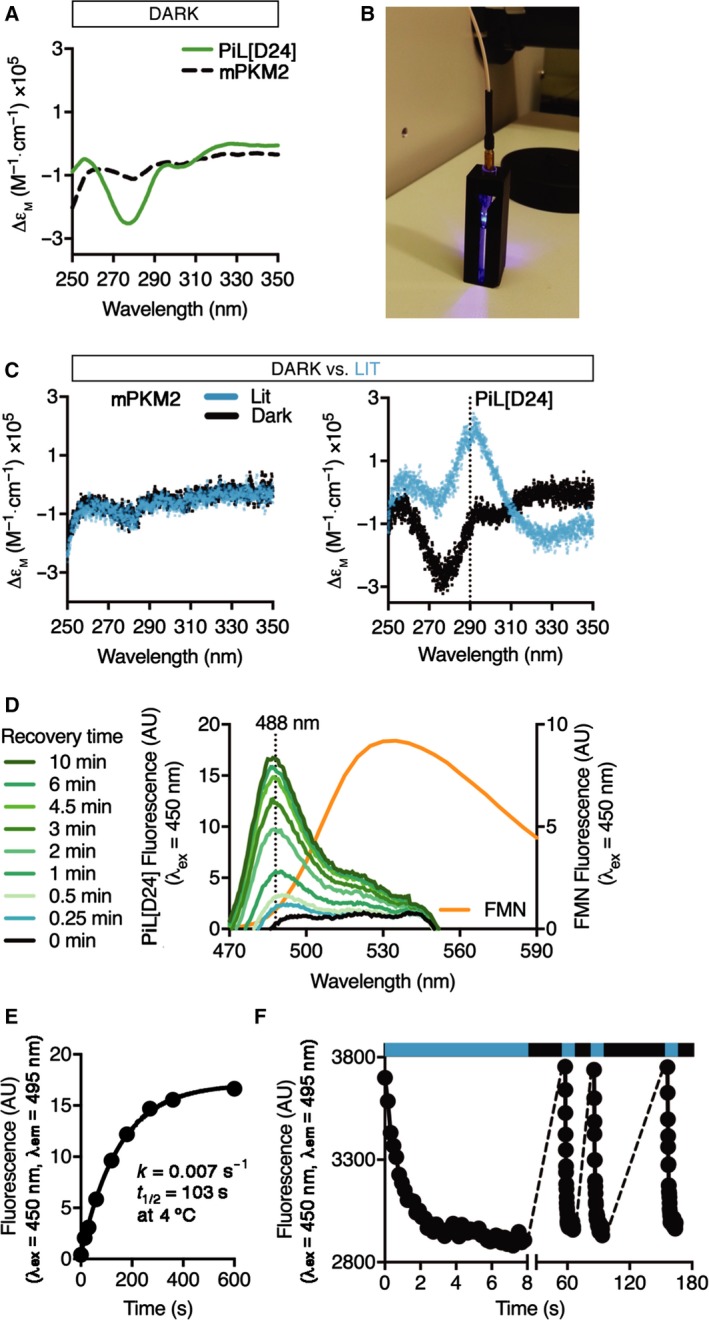
PiL[D24] is fluorescent and undergoes a reversible photoreaction. (A) Near‐UV CD spectra of purified recombinant PiL[D24] compared to mPKM2 showing a characteristic strong negative band at 278 nm for FMN. ∆ε_m_, molar extinction coefficient. (B) Setup for sample illumination in CD experiments. The optic fibre was positioned with a cut pipette tip on top of the sample in the quartz cuvette and were placed in the instrument. (C) Overlay of consecutive single near‐UV CD spectral scans of mPKM2 (left panel) and PiL[D24] (right panel) before (Dark) and after (Lit) repeated blue light illumination for 2 min (*n* = 5). (D) Fluorescence emission spectra of purified recombinant PiL[D24] at 4°C obtained by consecutive emission scans at the indicated recovery time following blue light (λ_ex_ = 450 nm) illumination. The dotted line at 488 nm marks the emission maximum of PiL[D24]. For comparison, the emission scan of free FMN, also at λ_ex_ = 450 nm is shown in orange. (E) Fluorescence at the emission maximum (488 nm) measured in (D) was plotted against time and showed a first‐order exponential reaction with *k* = 0.007 s^−1^ and *t*_1/2_ = 103 s. (F) Repeated photoswitching of purified recombinant PiL[D24] observed at its emission maximum (495 nm, λ_ex_ = 450 nm). Blue stripe highlights light exposure and black stripe no light.

We first addressed whether LOV2 inserted into PKM2 can bind to FMN and undergo photocycling. To this end, we used circular dichroism (CD) spectroscopy, which allows measurement of both the chromophore in near‐ultraviolet (UV) wavelengths (250–350 nm) and protein secondary structure in far‐UV (200–250 nm), in parallel 46, 47. The near‐UV CD spectrum of PiL[D24] in the Dark (no light) state is dominated by a strong optically active negative band at 275 nm that corresponds to FMN and is absent in the mPKM2 spectrum (Fig. 4A). Using an optic fibre to deliver blue light (460 nm) from a light‐emitting diode (LED) light source, we excited PiL[D24] directly in the spectrometer cuvette (Fig. 4B) and observed the emergence of a positive peak at 290 nm only with PiL[D24], which results from the light‐induced formation of the FMN‐cysteinyl adduct that creates a new chiral centre (Fig. 4C) 47. We corroborated these results by measuring the fluorescence emission spectrum of PiL[D24] at the maximum excitation wavelength for LOV2 (450 nm). Recombinant PiL[D24] showed the distinct fluorescence emission profile of protein‐associated FMN (Fig. 4D) that has a blue‐shifted emission maximum at 490 nm compared to free FMN (λ_em_ max = 530 nm) 48, 49, 50. Continuous excitation at 450 nm resulted in a decrease of fluorescence intensity to background levels. Following light withdrawal, PiL[D24] fluorescence at 488 nm recovered with first‐order kinetics (Fig. 4E) similar to LOV2 domain alone 46. Furthermore, PiL[D24] fluorescence recovered to the original intensity prior to illumination even after several photocycles (Fig. 4F). Together, the near‐UV CD and fluorescence experiments show that PiL[D24] copurifies with FMN, which is found in *E. coli* at μm concentrations 51; they also provide evidence that, in the context of PiL[D24], LOV2 retains its photoswitching properties and can undergo consecutive photoswitching cycles without loss of this functionality.

**Figure 5:**
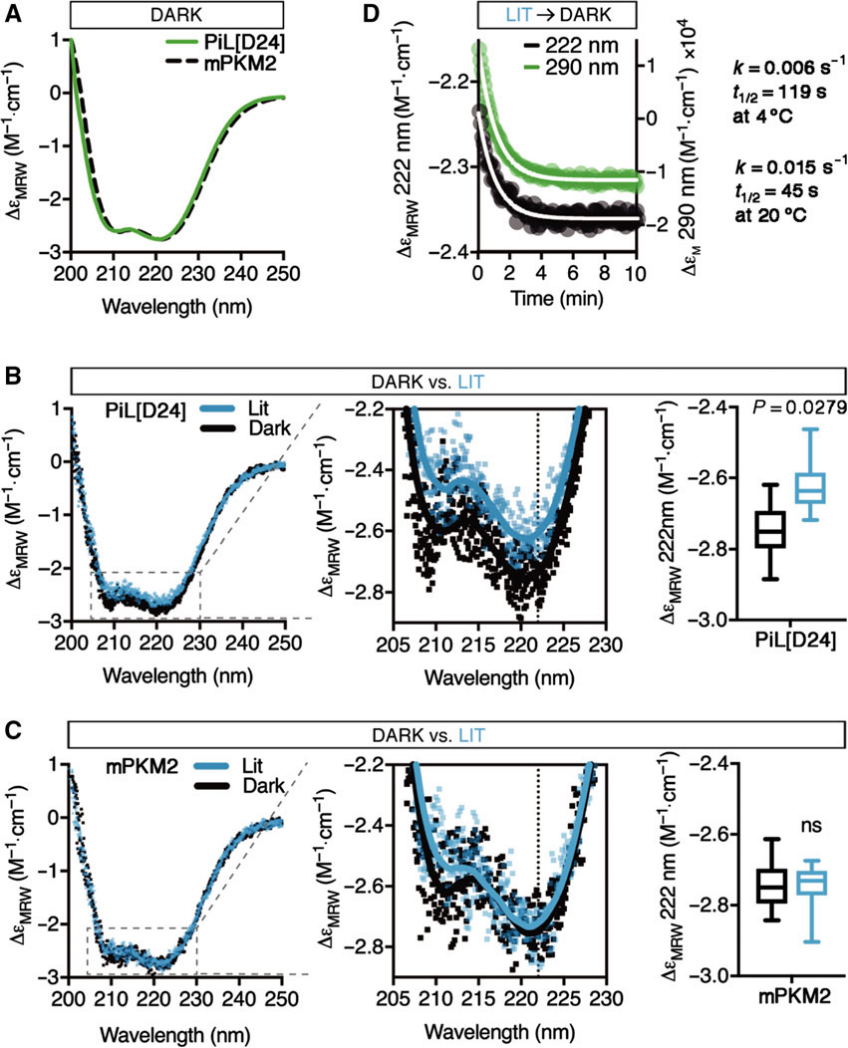
Light induces conformational changes in PiL[D24] that are synchronous to changes in its fluorescence. (A) Far‐UV CD spectra of purified recombinant PiL[D24] compared to mPKM2 showing indistinguishable secondary structure profiles that are characteristic of α‐helix‐rich proteins. ∆ε_MRW_: extinction coefficient of MRW. (B) Overlay of consecutive single far‐UV CD spectral scans of PiL[D24] before (Dark) and after (Lit) continuous blue light illumination for 2 min, *n* = 5. Zoom‐in highlights loss of α‐helical CD signal under the Lit condition. Solid lines represent the smoothed average of single scans. The dotted line at 222 nm marks the α‐helical signature CD peak where we observed a significant loss of signal (*P* = 0.0279, unpaired parametric *t*‐test) under the Lit condition, as shown on the adjacent bar graph. (C) Overlay of consecutive single far‐UV CD spectral scans or mPKM2 as in (B), acquired and quantified as in (B), *n* = 5 (*P* = 0.2320, unpaired parametric *t*‐test). Zoom‐in shows no loss of CD signal for mPKM2 under Lit condition. (D) Kinetics of secondary structure recovery measured by the CD signal at 222 nm (black circles, right *y*‐axis) compared to the kinetic of the FMN photoreaction recovery measured at 290 nm (green circles, left *y*‐axis) after 2 min of blue light exposure at 20 °C. Both signals recover with identical first‐order kinetics (*k* = 0.015 s^−1^ and *t*_1/2_=45 s).

The FMN photoreaction triggers the dissipation of the Jα helix at the C terminus of LOV2, which acts as a lever for signal communication 36, 37, 52. To investigate whether the light‐induced changes observed in the near‐UV spectrum of FMN are accompanied by structural changes in PiL[D24], we compared the far‐UV (200–250 nm) wavelengths of the CD spectra in the Dark and Lit states. Figure 5A shows that, in the Dark state, the PiL[D24] spectrum was indistinguishable from that of PKM2 and comprised two negative bands at 208 and 222 nm which are characteristic of α‐helix‐rich proteins. This is consistent with the predicted secondary structure content of the constituent proteins combined, both of which have an α + β fold (Table 1). In the Lit state, we observed a significant decrease in the CD signal of PiL[D24] (4.5 ± 2.4% at 222 nm; Fig. 5B). In contrast, the spectrum of PKM2 remained unchanged (‐0.2 ± 2.3%) between the Lit and Dark states (Fig. 5C). Given the coincidence of FMN and secondary structure changes, we compared the kinetics of the two reactions after illumination by monitoring dynamically the CD signal intensity at 290 nm and 222 nm, respectively, and found that they both followed identical first order exponential decay kinetics synchronously with a half‐life of 44 s at 20 °C, comparable to previously reported values for LOV2 (45–47 s) 47 (Fig. 5D). LOV2 and PKM2 have a similar α‐helical content (Table 1), so the fact that the PKM2 and PiL[D24] CD spectra overlap indicates that the secondary structure of the constituent proteins is largely preserved in the context of PiL[D24]. Furthermore, the far‐UV CD spectrum of α + β proteins is predominated by the α‐helical component 53, 54. Although CD does not allow the necessary resolution to determine which part of the protein undergoes conformational changes, these results are consistent with a loss of α‐helical content on functioning LOV2 after illumination, likely due to dissipation of the Jα‐helix as previously reported 36, 39, 40. Collectively, these experiments show that PiL[D24] undergoes light‐dependent conformational changes and that these changes can be inferred from measuring the fluorescence of PiL[D24].

**Table 1:** Calculated secondary structure content for LOV2 and hPKM2 calculated from the respective crystal structures, and estimated secondary structure content for PiL[D24]. hPKM2 was used for this calculation as no available crystal structures for mPKM2 exist

	α‐helical content (%)	β‐sheet content (%)	PDB‐file	Reference
LOV2	36	29	2V0U	52
hPKM2	36	19	3BJT	22
PiL[D24] estimated	35	20	–	–

### Light induces a reversible increase in the enzymatic activity of purified PiL[D24]

**Figure 6:**
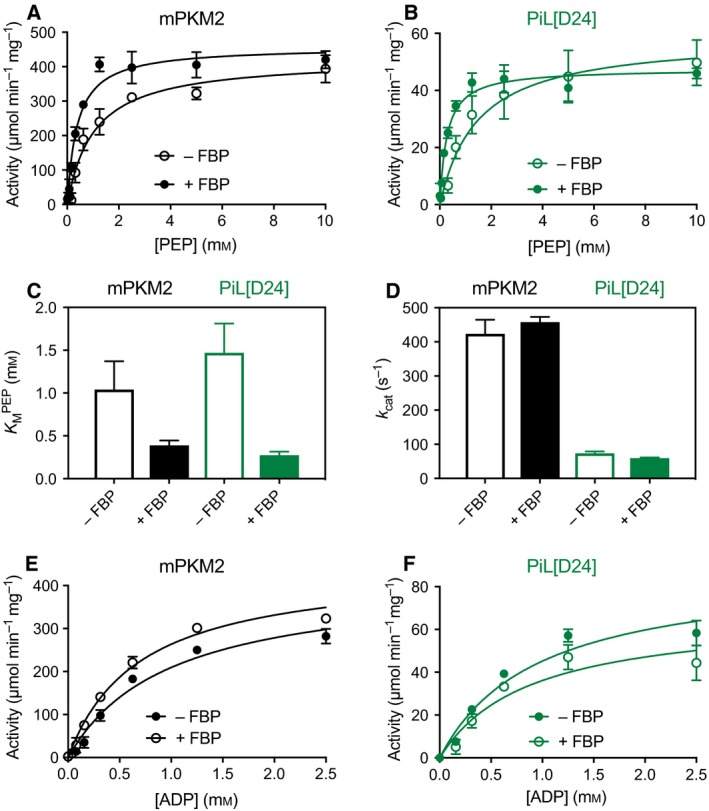
PiL[D24] has lower activity in the Dark state than mPKM2, but maintains the capacity to be activated by FBP. (A) Steady‐state enzyme kinetics of purified mPKM2 at varying concentrations of phosphoenolpyruvate. Initial velocity curves were fitted using Michaelis–Menten kinetics. For all phosphoenolpyruvate concentrations, ADP was added at a constant concentration of 2.5 mm in the absence (open circles) or presence (closed circles) of 400 μm FBP. (B) Steady‐state enzyme kinetics of purified PiL[D24] as in (A). (C) Plotting of the measured *K*_M_^PEP^ from (A) and (B) showing the effects of FBP addition on mPKM2 and PiL[D24]. (D) Comparison of the *k*_cat_ for mPKM2 and PiL[D24] measured from (A) and (B). (E) Steady‐state enzyme kinetics of purified mPKM2 at varying concentrations of phosphoenolpyruvate (ADP). Initial velocity curves were fitted using Michaelis–Menten kinetics. For all ADP concentrations, phosphoenolpyruvate was added at a constant concentration of 10 mm in the absence (open circles) or presence (closed circles) of 400 μm FBP. (F) Steady‐state enzyme kinetics of purified PiL[D24] as in (E). In all plots, the mean of three technical replicates is plotted and error bars indicate the standard deviation of the mean.

The overall fold, domain structure and catalytic site of pyruvate kinases have been conserved over millions of years of evolution, so the insertion of LOV2 could disrupt the enzymatic function of PKM2, despite the lack of major interference with secondary structure seen by CD. We therefore compared the activity of PiL[D24] to that of mPKM2 (Fig. 6 and Table 2) with a commonly used method that couples the PK reaction to lactate dehydrogenase (LDH) and monitors loss of nicotinamide adenine dinucleotide (reduced) (NADH) fluorescence (λ_ex_ = 340 nm, λ_em_ = 470 nm). In this assay, the *K*_M_ of mPKM2 for phosphoenolpyruvate was 1.04 ± 0.33 mm and the *k*_cat_ was 423.5 ± 41.3 s^−1^ (Fig. 6A). Under the same conditions, PiL[D24] showed a lower activity that reflected both a lower affinity for phosphoenolpyruvate (*K*_M_ = 1.47 ± 0.34 mm) and a lower *k*_cat_ (73.5 ± 5.6 s^−1^; Fig. 6B). Addition of FBP decreased the *K*_M_ of mPKM2 for phosphoenolpyruvate to 0.39 ± 0.06 mm with minimal effects on the *k*_cat_ (Fig. 6C–D). Similarly, FBP decreased the *K*_M_ of PiL[D24] for phosphoenolpyruvate to 0.27 ± 0.04 mm, which was similar to that for mPKM2 + FBP, indicating that the ability of FBP to activate the enzyme is preserved in PiL[D24]. Conversely, the *K*_M_ for ADP was similar between mPKM2 (0.98 ± 0.28 mm) and PiL[D24] (0.95 ± 0.37 mm), and did not change significantly with saturating FBP (Fig. 6E–F). The lower activity of PiL[D24] compared to mPKM2 suggests that the LOV2 insertion interferes with HLH conformation or dynamics and lends further credence to our hypothesis that the HLH is functionally linked to the active site. These data show that PiL[D24] retains enzymatic activity, albeit with lower catalytic efficiency than mPKM2.

**Table 2:** Steady‐state Michaelis–Menten kinetic parameters for mPKM2 and PiL[D24] under Dark conditions determined by an LDH‐coupled pyruvate kinase at 37 °C

	*k*_cat_ (s^−1^)		$K_{M}^{\mathrm{PEP}}$ (mm)		$K_{M}^{\mathrm{ADP}}$ (mm)	
	mPKM2	PiL[D24]	mPKM2	PiL[D24]	mPKM2	PiL[D24]
− FBP	423.5 ± 41.3	73.5 ± 5.6	1.04 ± 0.33	1.47 ± 0.34	0.98 ± 0.28	0.95 ± 0.37
+ FBP (400 μm)	457.5 ± 15.7	59.5 ± 21	0.39 ± 0.06	0.27 ± 0.04	0.69 ± 0.11	0.87 ± 0.40

**Figure 7:**
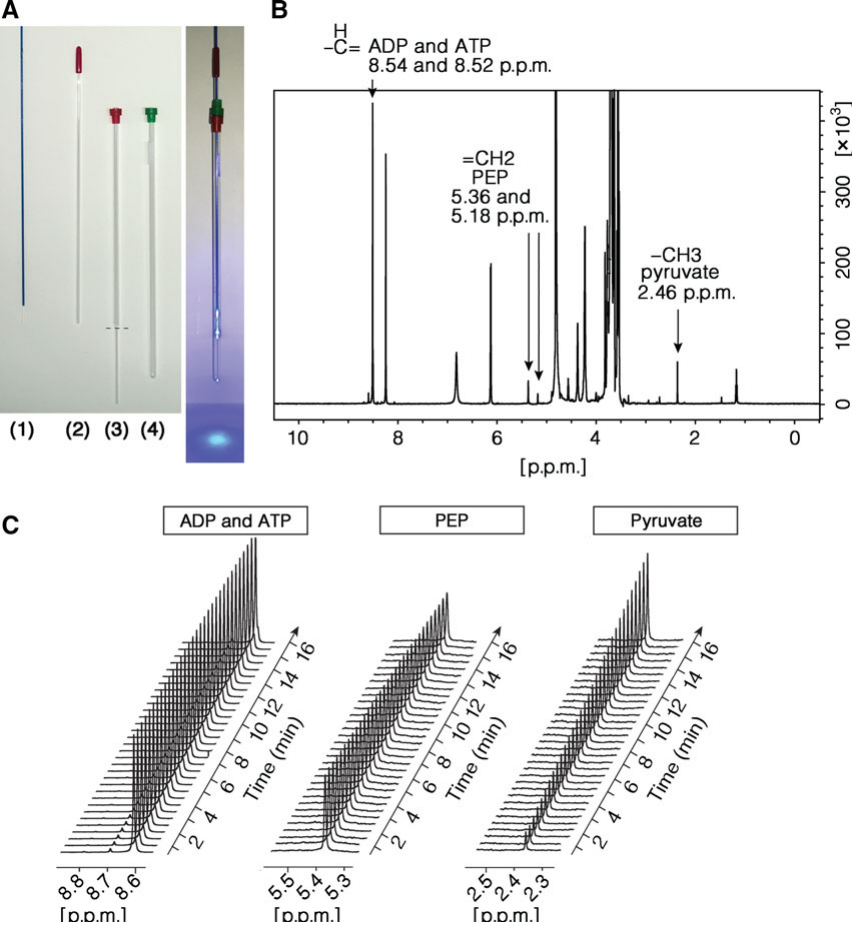
Monitoring of the pyruvate kinase reaction in real time by NMR. (A) Setup of optic fibre to illuminate the sample in the NMR tube (right picture). The fibre (1) is inserted into an inner tube (2) that is then placed into the coaxial insert (3) with its stem cut off (dotted line). This holds the fibre at the centre of the NMR tube (4). The fully assembled setup with the fibre illuminated is shown on the right picture. (B) 1D ^1^H NMR spectrum of reactants acquired in Tris/HCl buffer referenced to H_2_O/D_2_O and acquired with water suppression. Assigned hydrogens from pyruvate, phosphoenolpyruvate, ADP and ATP are indicated with arrows. (C) The time evolution of pyruvate kinase reaction shown by staged peaks for ADP and ATP, phosphoenolpyruvate and pyruvate extracted from sequential NMR spectra (as in B) that were acquired at 30‐s intervals.

**Figure 8:**
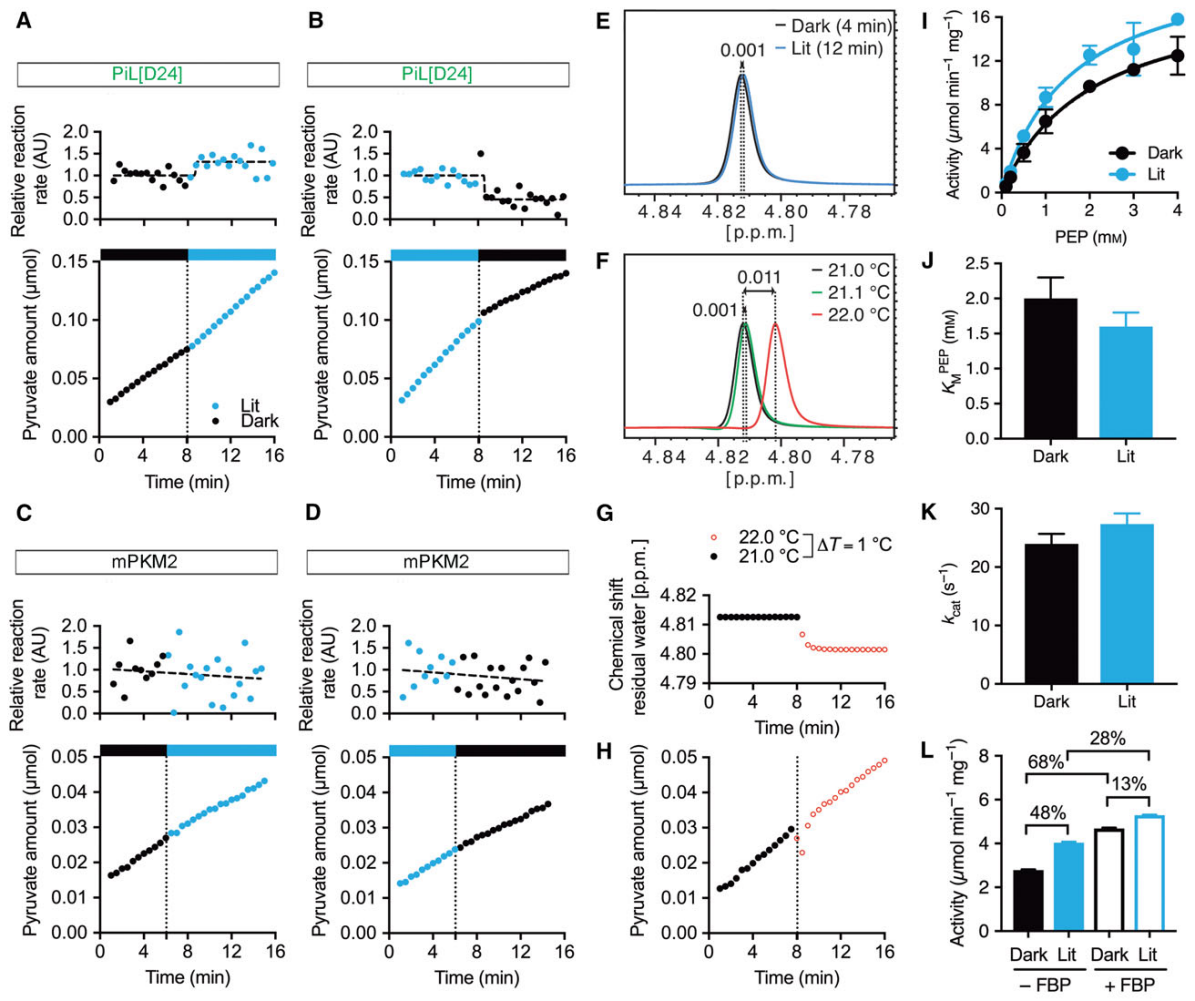
Light induces a reversible increase in the enzymatic activity of PiL[D24]. (A) Pyruvate production in PiL[D24] activity assay, quantified from a time‐resolved series of ^1^H NMR spectra, before (0–8 min, Dark, black stripe) and after (8–16 min, Lit, blue stripe) switching the blue light on (dotted line at 8 min). The top panel shows reaction rates over time and demonstrates increased activity in the Lit state. (B) Pyruvate production in PiL[D24] activity assay as in (A) but with inverse light conditions, starting the reaction under Lit conditions and switching the light off (Dark). Time‐dependent slopes demonstrate a decrease of activity for withdrawing blue light. (C) Pyruvate production in mPKM2 activity assay as in (A). (D) Pyruvate production in mPKM2 activity assay as in (B). (E) Water peaks from NMR spectra, at 4 min and 12 min, of the experiments shown in (A), showing a change in chemical shift of −0.001 p.p.m. after illumination. See Methods for details on calculating temperature change from the residual water resonance peak shift. (F) NMR spectra of water in the PK reaction buffer without enzymes at the indicated temperatures. (G) Temperature‐dependent change in the chemical shift of water in a PiL[D24] reaction, shown in (H), at 21.0 °C (0–8 min, black filled circles) and after the temperature was increased to 22.0 °C (8–16 min, red open circles). (H) Pyruvate production in PiL[D24] activity assay, quantified from a time‐resolved series of ^1^H NMR spectra under Dark conditions. The reaction started at 21.0 °C (black filled circles) and progressed for 8 min, at which point (dotted line) the temperature in the NMR tube was increased to 22.0 °C (red open circles). (I) Michaelis–Menten curve fittings (solid lines) of PiL[D24] activity measured with various concentrations of phosphoenolpyruvate ranging from 0.1 to 4 mm under in Lit and Dark state (mean ± SD, *P* = 0.0059, paired parametric *t*‐test, *n* = 3–4 activity measurements for each phosphoenolpyruvate concentration). (J) Bar graph representation of the $K_{M}^{\mathrm{PEP}}$ (mean ± SD) calculated from data shown in (E). Also see Table 3. (K) Bar graph representation of the *k*_cat_ (mean ± SD) calculated from data shown in (E). Also see Table 3. (L) Activity of PiL[D24] under Dark and Lit conditions in the absence or presence of 100 μm FBP with 1 mm phosphoenolpyruvate (value ± error of the linear fit of activity progress curves from three technical replicates). Percentages show change in activity between the corresponding conditions.

We next investigated whether the light‐induced changes in secondary structure seen by CD were accompanied by changes in the enzymatic activity of PiL[D24]. To this end, we developed an orthogonal analytical method to measure PK activity continuously, using ^1^H nuclear magnetic resonance (NMR) spectroscopy. We used an LED light source to deliver blue light via an optic fibre to the sample directly into the NMR tube (Fig. 7A). We first validated this approach by measuring PiL[D24] activity in the Dark, where we observed a linear increase in the spectral peak corresponding to the methyl group hydrogens of pyruvate (Fig. 7B–C). To test the effects of light, we allowed the reaction to proceed for 8 min, at which point we illuminated the sample and observed an increase in the rate of pyruvate production, indicative of an increase in enzymatic activity (Fig. 8A). We performed the same assay starting with an illuminated sample and found that the slope of the reaction curve was similar to the Lit state in Fig. 8A, while, upon light withdrawal, the activity decreased to basal levels (Fig. 8B). We did not observe light‐induced changes in the rate of pyruvate production by mPKM2 subjected to the same Dark–Lit or Lit–Dark regimes (Fig. 8C,D respectively). Furthermore, using the water resonance frequency as a proxy for the temperature in the sample, we determined that light increases the temperature in the reaction by < 0.1 °C (Fig. 8E–F). A deliberate increase of 1 °C in the temperature of the reaction under Dark conditions does not change the rate of pyruvate production by PiL[D24] (Fig. 8G–H) refuting the possibility that the increased activity with light is due to a light‐induced increase in the temperature of the reaction. These observations indicated that light increases the activity of PiL[D24] in a reversible manner.

To explore the basis of light‐induced increase in activity, we measured PiL[D24] enzymatic activity under Lit and Dark conditions over a range of phosphoenolpyruvate concentrations. The *K*_M_ for phosphoenolpyruvate obtained with this assay under Dark conditions (2.0 ± 0.3 mm, Table 3) was comparable to that from the coupled assay (1.47 ± 0.34 mm), albeit higher, likely because of the lower temperature used for the NMR assay (ambient, 21 °C) compared to the coupled assay (37 °C). We observed a reproducible decrease in the *K*_M_ for phosphoenolpyruvate under Lit compared to Dark (Fig. 8I–J, Table 3) conditions. Intriguingly, the *k*_cat_ of the reaction under Lit conditions also showed a trend to increase (Fig. 8K, Table 3). This is reminiscent of a phenomenon seen with PKM2 activators 55, 56, which bind to the HLH at a pocket proximal to the LOV2 insertion site 13. In the presence of saturating FBP, PiL[D24] activity increased 13% after illumination, compared to 48% without FBP (Fig. 8L), supporting our starting hypothesis that the selected LOV2 insertion site is involved in FBP‐induced allostery. Together these data showed that light increases the activity of PiL[D24], in a reversible manner, by promoting a higher affinity for its substrate phosphoenolpyruvate.

**Table 3:** Steady‐state Michaelis–Menten kinetic parameters for PiL[D24] under Dark and Lit conditions determined by NMR spectroscopy at 21 °C

	*k*_cat_ (s^−1^)	$K_{M}^{\mathrm{PEP}}$ (mm)
Dark	23.99 ± 1.70	2.03 ± 0.30
Lit	27.39 ± 1.79	1.42 ± 0.23

### Light modulates PiL[D24] activity in cells

**Figure 9:**
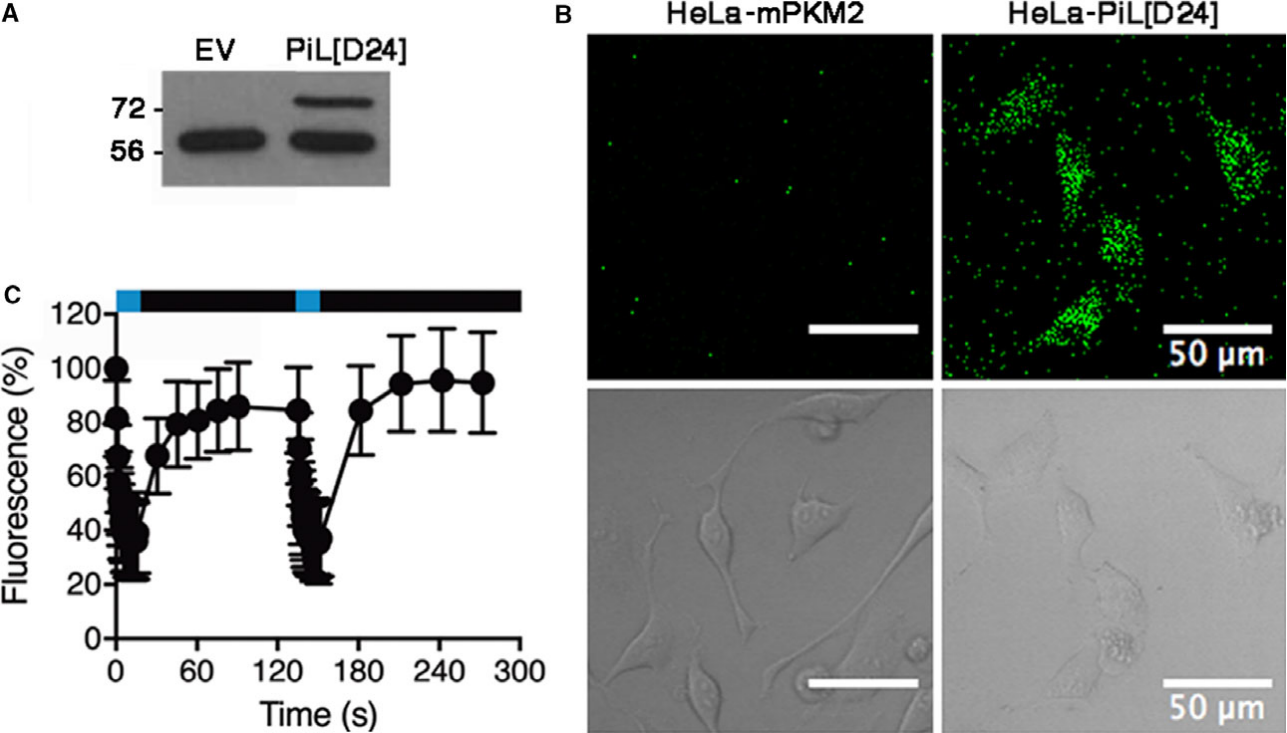
PiL[D24], expressed in HeLa cells, undergoes photoswitching. (A) Western blot of lysates from control empty vector (EV) or PiL[D24]‐expressing HeLa cells, probed with a PKM2 antibody, revealed a higher molecular weight band, which is only present in HeLa‐PiL[D24] cells and corresponds to the expected size for PiL[D24] (75 kDa) compared to the endogenous hPKM2 (lower band). (B) LOV2 fluorescence (upper panel) and transmitted light images (lower panel) in cultured HeLa cells expressing PiL[D24] in comparison to mPKM2 expressing HeLa cells. (C) Fluorescence switching in HeLa‐PiL[D24] cells demonstrates loss of fluorescence under fast acquisition (1 frame/0.6 s) and recovery under sparse (1 frame/15 s and 1 frame/30 s) imaging regimes (*n* = 36, mean ± SD). Blue stripe highlights light exposure and black stripe no light in the imaging time course.

Forced increases in pyruvate kinase activity can influence proliferative cancer metabolism, so we wanted to provide proof‐of‐principle that PiL[D24] could be useful for experiments in cells. We used retroviral transduction to express PiL[D24] stably in HeLa cells in the presence of endogenous PKM2 (Fig. 9A). These cells are henceforth referred to as HeLa‐PiL[D24]. We could visualise the subcellular localisation of PiL[D24], due to its fluorescence, by confocal microscopy imaging, which revealed a cytoplasmic expression, whereas no fluorescence was observed in control cells transduced with virus that expressed mPKM2 (Fig. 9B). PiL[D24] fluorescence decreased rapidly after illumination with blue light (Fig. 9C), similar to purified protein *in vitro*, and the kinetics of fluorescence recovery were identical to those observed with purified protein (*k* = 0.015 s^−1^ for both). These data confirm that light can modulate the photoreaction of PiL[D24] expressed in mammalian cells, where the protein is likely subject to modifications that are not present in bacterially expressed protein.

**Figure 10:**
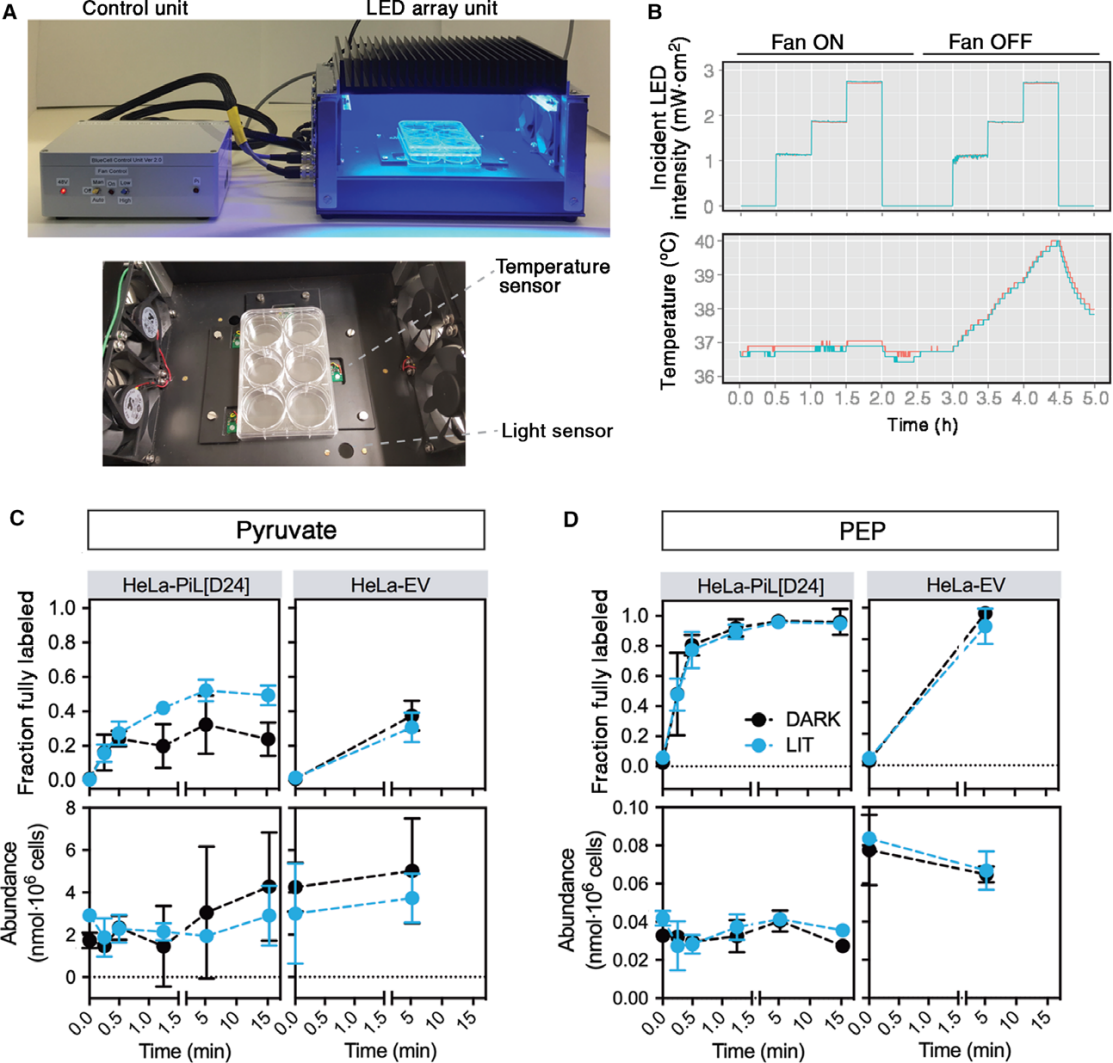
Blue light increases pyruvate kinase activity in cells expressing PiL[D24]. (A) Picture of the BlueCell unit (top) and of a plate places within the unit (bottom), which we constructed for illumination of cultured cells. BlueCell is based on an LED light array that can be placed within a humidified CO_2_ incubator and is operated via a raspberry pi in the control unit (upper panel, grey box). The led array has the capacity to deliver continuous or pulsed light with variable intensity (1–10 mW·cm^−2^). Throughout the experiment, the incident light experienced by cells and the actual temperature in the LED unit chamber are monitored with optical and temperature sensors that are embedded next to the cell culture plate (bottom image). Integrated fans can be used as needed to dissipate the heat generated by the LEDs. (B) Example of measured light intensity and temperature output showing increased temperature in the chamber due to LED heating which increases with higher light intensities. Use of the embedded fans (‘fan ON’) alleviates this effect and allows the maintenance of a stable temperature. The two traces indicate readings from two separate sensors within the unit. (C) Fraction of pyruvate that is fully labelled from U‐^13^C‐glucose (upper panel) and pyruvate abundance (lower panel) in control (HeLa EV) and HeLa‐PiL[D24] cells. Cells were seeded in replicates (four for HeLa‐PiL[D24], three for HeLa EV) at *t* = −18 h in six‐well plates and cells were preilluminated for 1 h (*t* = −1 h) for Lit condition or left in the Dark. U‐^13^C‐glucose was then added to the media (*t* = 0 min) while the Dark or Lit conditions were maintained for the respective groups. Cells were quenched and harvested at the indicated time points. The polar fractions of the respective cell extracts were analysed by GC‐MS to quantify ^13^C labelling and total abundance of pyruvate (mean ± SD). (D) Fraction of phosphoenolpyruvate that is fully labelled from U‐^13^C‐glucose (upper panel) and phosphoenolpyruvate abundance (lower panel) in control (HeLa EV) and HeLa‐PiL[D24] cells as described in (C). Raw natural abundance‐corrected GC‐MS data are shown in Table S1.

To test whether blue light can activate PiL[D24] in intact cells, we incubated HeLa‐PiL[D24] and control cells with media containing universally labelled ^13^C glucose ([U‐^13^C]‐glucose) and monitored the rate of ^13^C incorporation into pyruvate using gas chromatography–mass spectrometry (GC‐MS). For homogenous illumination of cells with blue light, we constructed an LED array device with embedded temperature sensors and a cooling system that bestows the capacity to maintain stable temperature (37 ± 0.2 °C; Fig. 10A–B) to minimise light‐induced temperature fluctuations that could influence cellular metabolic rates. In the dark, incorporation of ^13^C into pyruvate reached isotopic steady‐state within 5 min with similar enrichment (~ 35%) in both cell lines under Dark conditions (Fig. 10C and Table S1). During the course of the experiment, total pyruvate levels remained largely unchanged. Illumination with blue light caused an increased labelling of pyruvate to 52 ± 6% (at 5 min) only in HeLa‐PiL[D24] cells, whereas label incorporation into phosphoenolpyruvate was unchanged (Fig. 10D). In conclusion, these data show that light increases PiL[D24] activity in cells in the background of endogenous PKM2.

## Discussion

**Figure 11:**
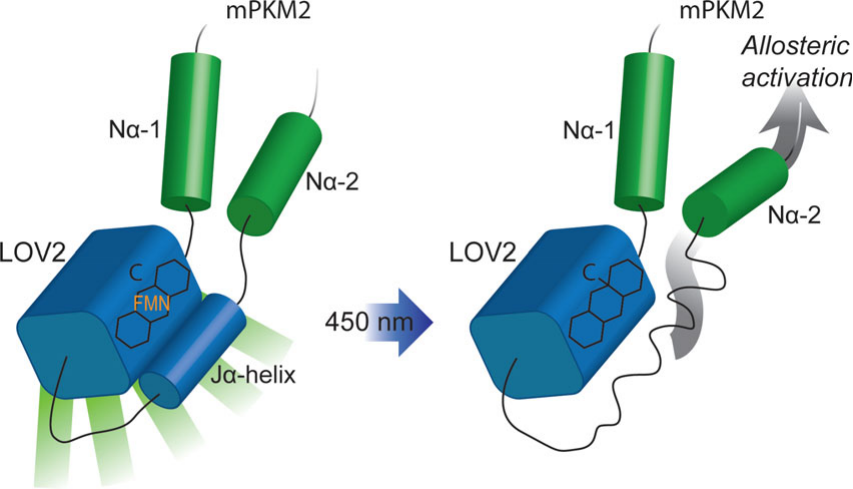
Proposed model for the light‐dependent activation of PiL[D24]. In the Dark state (left), PiL[D24] fluoresces (denoted by the green bars emanating out of the LOV2 domain). Light induces a conformational change in LOV2 that is transmitted (grey arrow on the right cartoon) to the flanking PKM2 part of the chimera, leading to an increase in its pyruvate kinase activity. Light activation leads to the loss of PiL[D24] fluorescence. C: cysteine in the LOV2 PAS domain; shown forming a cysteinyl adduct with FMN on the right.

In this study, we showed that insertion of the photosensory LOV2 domain into mPKM2 at a site that undergoes conformational changes in response to its endogenous activator FBP allows the control of PKM2 enzymatic activity with light *in vitro* and in cells. Based on our data, a proposed model summarising how light may modulate PiL[D24] activity is shown in Fig. 11. PiL[D24] retained PK activity, although this was lower than the native mPKM2 indicating that there is a trade‐off between the capacity to modulate activity with LOV2 through a pre‐existing mechanism and the preservation of intact enzymatic activity. While this could be a hindrance for other enzymes, the fact that PKM2 activity is kept low to support proliferation permits the use of PiL[D24] in cells. Notably, the magnitude of light‐induced activity increase with purified PiL[D24] (up to 48%, Fig. 8L) is comparable to the changes in the activity of other enzymes rationally engineered with domain insertions for allosteric control 57, including previously reported LOV2‐enzyme chimeras 41, 43.

Changes in PKM2 activity in response to endogenous cellular signalling cues range from 10% to 50% and mutations in PKM2 or activating compounds that abrogate these regulatory mechanisms impede the ability of PKM2 to support proliferation and survival of cancer cells 13, 14, 21, 22, 23, 24, 58. In this context, despite the relatively modest effect of light on its activity, PiL[D24] may prove useful in studying the functional consequences of acute or chronic PKM2 activation on cellular physiology. It is likely that endogenous cellular mechanisms that regulate PKM2 can influence the magnitude of the effects of LOV2 on PKM2 activity. We could show that light leads to a detectable increase in pyruvate labelling from glucose, even when PiL[D24] is expressed at substoichiometric levels compared to endogenous PKM2 (Fig. 9A). This indicates that light‐induced changes in PiL[D24] activity can overcome potential endogenous regulatory mechanisms that normally maintain PKM2 in a low‐activity state. Conversely, PiL[D24] can still be activated by FBP (Fig. 6B–C), even under Lit conditions (Fig. 8L) suggesting that the presence of LOV2 does not disrupt the endogenous allosteric mechanism and raising the possibility that additional, redundant allosteric pathways exist. As little is known about the mechanical basis of PKM2 regulation by FBP and other ligands, either independently, or in combination, PiL[D24] will be useful in elucidating the molecular basis of PK allostery.

### Rational design of LOV2‐based optogenetics tools for metabolism

The use of LOV2 in cellular optogenetics has led to invaluable insights into various aspects of cellular physiology 59, 60, 61 as it provides a means to control protein function specifically, reversibly and in a spatially and temporally resolved manner that is inaccessible to other techniques. However, no such tools exist to modulate metabolism in mammalian cells. Many optogenetic tools are based on fusions of light‐sensory domains at the termini of target proteins and their functionality relies on modulating protein–protein or intermolecular interactions 39. This can be difficult to implement with proteins whose activity relies on the formation of higher order (more than two subunits) oligomers.

Internal fusions of LOV2 at structurally dynamic sites that have functional roles within target proteins can exploit the conformational changes of the LOV2 photocycle to modulate protein activities that rely on mechanical motion. A major challenge with this approach is the selection of suitable insertion sites. When protein function can be coupled to a measurable phenotype, such as cell proliferation or viability, systematic screens are advantageous as they readily provide a functional validation of the optogenetic tool. However, such screens can be laborious in mammalian cells, so alternative approaches for guiding the design of engineered LOV2‐target protein chimeras are desirable. Computational methods to investigate allosteric mechanisms have proven invaluable as they can probe protein conformational dynamics at relevant time‐scales 62. In our study, we show that insights into protein allostery gained from MD simulations can be used to identify potential LOV2‐regulatable insertion sites. Our strategy relied on the assumption that structural dynamics participate in the relay of allosteric information between the FBP and catalytic pockets. The estimation of free energy changes provided an independent means to validate whether the observed conformational dynamics seen in the MD trajectories of hPKM2‐FBP are involved in allostery. However, there are instances where allosteric communication is not accompanied by gross mechanical motions 28, 63, 64. We cannot exclude the possibility that allosteric mechanisms that do not involve conformational changes exist in PKM2; however, our results suggest that the conformational dynamics of the N‐terminal HLH drive, at least in part, the allosteric effect of FBP on PKM2 activity. As our understanding of the energetics of allostery evolves, the combination of enthalpic and entropic changes associated with light‐sensory domain photocycles may be harnessed in sophisticated ways to also modulate entropically driven allostery.

### Implications for the mechanism of PKM2 allostery and PKM2 activator mode of action

Interestingly, the LOV2 insertion site between Asp24 and Thr25 of PKM2 is proximal to the binding pocket of several distinct classes of PKM2 activators, which lead to increased PKM2 activity by lowering the *K*_M_ for phosphoenolpyruvate, i.e. in a manner that kinetically resembles the FBP mode of action. The activator pocket is distinct from any of the known binding sites of endogenous PKM2 allosteric ligands 17, 22, 65, so the structural basis of their mode of action has remained elusive. Asp24 does not make direct contacts with activators but resides at the loop linking the two N‐terminal α‐helices. The fact that these two helices undergo dynamic conformational changes upon FBP binding suggests that this region is involved in modulation of activity. Our data indicate that, similar to activators, LOV2 hijacks a pre‐existing allosteric pathway that relies on the N terminus of PKM2.

Our work demonstrates that allostery‐modulating optogenetics could provide a means to validate computationally predicted allosteric sites and complement conventional mutagenesis methods to guide targeted screens for small molecule allosteric modulators 66. Moreover, PiL[D24] offers a means to selectively engage the FBP allosteric mechanism and investigate how FBP binding influences the action of other ligands and vice versa. This can prove particularly valuable in cells, where selective addition or depletion of specific allosteric ligands is not possible.

### Outlook and applications of optogenetic tools in metabolism

Furthermore, PiL[D24] could provide a useful tool to probe the functional role of allostery‐mediated metabolic dynamics beyond cancer as pyruvate kinases play important roles throughout evolution and in a spectrum of physiological functions. In *E. coli*, the bacterial orthologue Pyk is an integral component of a reaction system that serves as a glycolytic flux sensor that allows the bacterium to respond to carbon availability 67. In both yeast and the mammalian liver, allosteric regulation of the respective PK isoforms by FBP and phosphorylation, respectively, precedes changes in protein expression to control the switch between glycolysis and gluconeogenesis 68, 69, 70. It is not clear whether, in these settings, the PK allosteric response is all‐or‐none, or whether dynamic fluctuations of PK activity influence particular functions. For example, in mammalian pancreatic β‐cells, PKM2 activity oscillates with a frequency of ~ 1 Hz 71, a behaviour that correlates with glycolytic oscillations 72. It has been argued that glycolytic oscillations are a systems design property rather than having evolved to perform a specific function 73. Conversely, glycolytic oscillations in pancreatic islets have been implicated in pulsatile insulin secretion, which is lost in diabetes, pointing to a functional role in maintaining organismal glucose homeostasis 74, 75. PiL[D24] could provide a means to modulate the glycolytic oscillator properties and experimentally test the functional significance of periodic phenomena in metabolism.

Finally, in addition to pyruvate kinases, altered allosteric properties of other glycolytic enzymes have been implicated in cancer 76, 77. A comprehensive understanding of the role of allostery in metabolism would require a systematic study of the respective role of all these enzymes. Optogenetic tools will likely prove invaluable in the quest to investigate how rapid allosteric phenomena influence metabolism.

## Methods

### Molecular dynamics simulations in explicit solvent

Explicit solvent MD simulations of monomeric human pyruvate kinase M2 (hPKM2) were performed and analysed using the gromacs 5.2 78 program using the Gromos 53a6 force field parameter sets 79. The input structure coordinates of apo‐hPKM2 were extracted from Protein Data Bank (PDB) ID 3BJT 22 and the structure of hPKM2 bound to the endogenous allosteric activator fructose‐1,6‐bisphosphate (FBP) was taken from PDB ID 3U2Z 13. The initial protein complex was solvated and minimised in a dodecahedral periodic box of explicit single point charge water molecules with an Ewald correction 80. A minimum distance of 1.0 nm was created between any protein atom and the periodic box. The system charge was neutralised by adding counter ions to the solvent. The equations of motions were integrated using the leapfrog method 81 with a 2‐fs time step. The equilibration protocol described in Schmidt *et al*. 82 was used. Briefly, the system was equilibrated in the canonical ensemble, followed by a 5‐ns NVT equilibration run at 300 K and 1 bar. This was further followed by 3 ns of equilibration in the NPT ensemble. After successful equilibration of the system, the apo‐PKM2 and PKM2‐FBP systems were simulated for 300 ns under constant pressure and temperature conditions, 1 bar and 300 K. Three replicas were performed for each system, giving a total simulation time of 1.8 μs. Temperature was regulated using the velocity‐rescaling algorithm 83, with a coupling constant (τ) of 0.1. All protein covalent bonds were frozen with the LINCS method 84, while settle 85 was used for water molecules. Electrostatic interactions were calculated with the particle mesh Ewald method 86, with a 1.4 nm cut‐off for direct space sums, a 0.12 nm FFT grid spacing and a four‐order interpolation polynomial for the reciprocal space sums.

### Analysis of molecular dynamics simulations

Molecular dynamics simulation trajectories were analysed using a combination of in‐house scripts and analysis modules within gromacs 5.2 78. To quantify local flexibility along the protein structure, the root‐mean‐square fluctuations (RMSF) over each Cα atom was computed as:

(1.1) $${\text{RMSF}}_{i}=\sqrt{\frac{\sum_{j}{\left(r_{i,j}-{\bar{r}}_{i}\right)}^{2}}{N}}$$

where *r*_*i*_ is the position of the Cα atom *i*, and *j* the time along the trajectory, following a least‐squares structural alignment to the starting structure.

Solvent accessibility was measured using the double cubic lattice method as described in Eisenhaber *et al*. 87. Briefly, the surface of each atom was approximated by creating a mesh of points and then surface accessibility was measured by counting the number of mesh points on the surface of the protein and not within the radius of a neighbouring atom.

After removing roto‐translational degrees of freedom, a PCA of the MD trajectories was performed from the covariance matrix of the atomic positional fluctuations with the elements

(1.2) $$\sigma_{ij}=〈\left(x_{i}-〈x_{i}〉\right)\left(x_{j}-〈x_{j}〉\right)〉$$

where *x*_*1*_ *…x*_*3N*_ are the Cartesian coordinates of an *N* particle system. The covariance matrix was then expressed in mass‐weighted coordinates

(1.3) $$\sigma^{\prime }=M\sigma$$

where *M* is the mass matrix of the protein. From the mass‐weighted covariance matrix, the eigenvalues and eigenvectors were extracted through a linear transformation in the form

(1.4) $$(\sigma^{\prime }-I\lambda )x=0$$

where *I* is the unit matrix. Solving the eigenvalue problem for the unknowns *x* and λ in Eqn (1.4) gives the eigenvectors and eigenvalues, respectively. In order to project the MD trajectories into a common principal component subspace, we applied the same rotation matrix to each coordinate system with equivalent atomic composition. This routine is implemented in an R script and is freely available from the authors upon request.

### Calculation of theoretical allosteric free energy

To calculate the predicted contribution of hPKM2 residues to endogenous allosteric activation of the protein, we used a statistical‐mechanical approach described by Guarnera and Berezovsky 27, extended for analysing thermodynamic models of protein dynamics from atomistic MD simulations. From exploring the conformational space accessible to PKM2 in the apo state, and when bound to the activator FBP, two sets of eigenvalues $\varepsilon_{\mu }^{P}$ and $\varepsilon_{\mu }^{PL}$ were obtained from the semiempirical force‐field protein model for the apo‐hPKM2 and hPKM2‐FBP simulations, respectively. These sets of eigenvectors are used as inputs for the calculation of the allosteric free energy:

(1.5) $$\Delta g_{i}\left(P\to PL\right)=\frac{1}{2}k_{B}T\sum_{\mu }\text{ln}\frac{\varepsilon_{\mu ,i}^{PL}}{\varepsilon_{\mu ,i}^{P}}$$

where ∆*g*_*i*_ is the per‐residue *i* free energy difference between the ligand‐bound and ligand‐free protein states, $\varepsilon_{\mu ,i}^{PL}$ is the μth eigenvalue for residue *i* in the PKM2‐FBP simulation and $\varepsilon_{\mu ,i}^{P}$ is the μth eigenvalue for residue *i* in the apo‐PKM2 simulation, *k*_B_ is the Boltzmann constant and *T* is temperature. The free energies calculated from Eqn (1.5) quantify the maximal configurational work ∆*g*_*i*_(*P→PL*) that is exerted on a residue *i* as a consequence of the change in the dynamics caused by ligand binding 27.

### Cloning of LOV2 into PKM2

To construct PiL[D24], the Gibson assembly cloning method was used 88. Three fragments coding for the N terminus of PKM2, LOV2 and the C terminus of PKM2 were amplified by polymerase chain reaction (PCR). LOV2 (residues 404–540) was amplified (primers SG034 and SG035) from plasmid pDS257 coding for Mid2(SS/TM)‐GFP‐LOVpep (a gift from Michael Glotzer, Addgene plasmid # 34971) 61. AsLOV2 positions are numbered as in phototropin 1 (NPH1‐1 GenBank: AAC05083.1) and G528A and N538E were backmutated stepwise by PCR to wild‐type LOV2. PKM2 residues 1–24 (primers SG032 and SG033) and residues 25–531 (primers SG036 and SG037) were amplified from pLHCX‐flag‐mPKM2 24 introducing *Eco*RI, *Hpa*I and *Cla*I, *Xho*I for further subcloning in addition to the 15‐nucleotide overlap with the N and C terminus of LOV2, respectively, for Gibson cloning. High fidelity Phusion polymerase was used for PCR. Fragments were assembled with the Gibson assembly cloning kit (NEB, #E5510S; NEB, Ipswich, MA, USA) and cloned into *Eco*RI/*Xho*I‐digested p413TEF using 0.08 pmol insert and 0.03 pmol vector in a total reaction volume of 20 μL to generate vector p413TEF‐*Eco*RI‐*Hpa*I‐PiL[D24]‐*Cla*I‐*Xho*I. The insert of p413TEF‐*Eco*RI‐*Hpa*I‐PiL[D24]‐*Cla*I‐*Xho*I was digested with *Hpa*I/*Cla*I and was subcloned into pLHCX (Clontech, Mount View, CA, USA) to generate vector pLHCX‐PiL[D24] used for retrovirus production. For recombinant His‐tagged protein expression in *E. coli*, PiL[D24] was PCR amplified (primers SG077 and SG037) from p413TEF‐*Eco*RI‐*Hpa*I‐PiL[D24]‐*Cla*I‐*Xho*I, to introduce an *Nde*I site at the 5′ of the fragment, digested with *Nde*I/*Xho*I and cloned into pET28a (Novagen, Darmstadt, Germany) to generate pET28a‐PiL[D24].

**Table:** 

Table of primers used in this study	
(lowercase = vector, uppercase = insert PKM2, uppercase and underlined = insert LOV2, lowercase and underlined = RE cleavage sites)	
SG032	gtggatcccccgggctgcaggaattcgttaacaccATGCCGAAGCCACACAGTGA
SG033	tccatgcagccatggctgacTTGGCTACTACACTTGAACG
SG034	TTGGCTACTACACTTGAACG
SG035	ATCAATATTTTCTGCAGTTT
SG036	AAACTGCAGAAAATATTGATACCTTCCTGGAACACATGTG
SG037	GTGTAGTGCCTGTACCTTGAatcgatctcgagtcatgtaattagttatgtca
SG077	cgccatatgccgaagccacacagtg

### Recombinant protein expression

pET28a‐PiL[D24] was transformed into *E. coli* BL21 (DE3) pLysS (60413; Lucigen, Middleton, WI, USA). Colonies were inoculated in LB medium at 37 °C, grown to 0.8 OD_600_ and expression of N‐terminal His_6_‐tag‐PiL[D24] was induced with 0.5 mm IPTG. The culture was then grown at 18 °C for 16–18 h. Cells were harvested by centrifugation and the pellet was resuspended in lysis buffer (50 mm Tris/HCl pH 7.5, 10 mm MgCl_2_, 200 mm NaCl, 100 mm KCl, 10 mm imidazole, 20% glycerol) supplemented with protease inhibitors [1 mm 4‐(2‐aminoethyl)benzenesulfonyl fluoride, 4 μg·mL^−1^ aprotinin, 4 μg·mL^−1^ leupeptin and 4 μg·mL^−1^ pepstatin (pH 7.4)], 2 mm dithiothreitol (DTT) and 0.8 mg·mL^−1^ lysozyme. After 1‐h incubation on ice, lysis was completed by three consecutive cycles of snap‐freezing in liquid N_2_ and thawing at 37 °C. DNase was added at 1 μL·mL^−1^ before the lysate was centrifuged at 20 000 ***g*** for 1 h at 4 °C. The supernatant was incubated with Ni‐nitrilotriacetic acid agarose (101844; Qiagen, Hilden, Germany) for 2 h at 4 °C on a rotating wheel and the resin was recovered on a column (9704652, Econo‐Pac Column; Bio‐Rad, Hercules, CA, USA) by gravity‐flow. The resin was washed in the column with lysis buffer containing 30 mm imidazole and His_6_‐tag‐PiL[D24] was eluted with 250 mm imidazole in lysis buffer. The affinity‐purified protein was further purified by size‐exclusion chromatography (SEC) on a HiLoad 16/600 Superdex 200 pg column (28‐9893‐35; GE Healthcare, Little Chalfont, UK) at 0.5 mL·min^−1^ flow rate with SEC‐buffer [50 mm Tris/HCl pH 7.5, 10 mm MgCl_2_, 200 mm NaCl, 150 mm KCl, 5% glycerol, 0.5 mm TRIS(2‐carbobylethyl)phosphine hydrochloride solution (TCEP)] monitoring absorbance at 280 nm. Fractions containing tetrameric His_6_‐tag‐PiL[D24] were combined and concentrated to 1–2 mg·mL^−1^ with centrifugal filters (Vivaspin 20, 10 kDa MWCO, 28‐9323‐60; GE Healthcare). Protein concentration was determined by the Bradford assay.

### Circular dichroism (CD) spectroscopy

Protein CD spectra were recorded using a JASCO J‐815 CD spectrometer. Full spectra from an average of 25 scans were recorded at 4 °C. Far‐UV CD spectra were recorded from 190 to 250 nm with 300 μL of 3.4 μm (0.26 mg·mL^−1^) PiL[D24] and 3.6 μm (0.2 mg·mL^−1^) PKM2 in a quartz cuvette with a path length of 0.1 cm. Near‐UV spectra were recorded from 250 to 350 nm with 500 μL of 13.3 μm (1 mg·mL^−1^) PiL[D24] and 17.1 μm (1 mg·mL^−1^) PKM2 in a quartz cuvette with a path length of 1 cm 46, 47. To continuously illuminate the sample at 460 nm prior to the measurement an optic fibre (FT200UMT, without tubing, end 1: FC/PC, end 2: flat cleaved, Thorlabs, Newton, MA, USA) coupled to a blue LED light source (UHP‐LED‐460 with an LED current controller; Prizmatix, Givat‐Shmuel, Israel) was placed on the surface of the sample in the cuvette and the CD spectrometer lid was covered with aluminium foil to prevent interference from ambient light. The sample was illuminated for 2 min (light intensity of ~ 3.3 mW·cm^−2^) to ensure complete photoswitching of PiL[D24]. Only a single scan was acquired (~ 20–30 s duration) to avoid recovery of the light‐induced state of PiL[D24]. Five single scans were recorded in a cumulative fashion by alternating five times between a scan of the 2‐min illuminated sample followed by a scan of the 3‐min recovered sample. Recovery kinetics over 10 min after 2‐min illumination were recorded at a wavelength of 222 nm (0.1 cm path length quartz cuvette, 300 μL volume) to follow the conformational change and at 290 nm (1 cm path length quartz cuvette, 500 μL volume) to follow the LOV2 photoreaction at 4 and 20 °C using 17.1 μm PiL[D24] and 15.8 μm PKM2. For far‐UV CD spectra, the raw data in mdeg were converted to extinction coefficient of the mean residue weight (MRW) 54:

$$\begin{array}{cc} \Delta \varepsilon \text{MRW}= & \frac{\text{CD Signal}\;(\text{mdeg})\times \text{MRW}}{\text{concentration}\left(\frac{\text{mg}}{\text{mL}}\right)\times \text{path length (cm)}} \end{array}$$

in (M^−1^cm^−1^), and for near‐UV CD spectra to the molar extinction coefficient:

$$\Delta \varepsilon_{M}=\frac{\text{CD Signal}\;(\text{mdeg})\times \text{MRW}}{\text{concentration}\;(M)\times \text{path length (cm)}}$$

$\text{in}\;(M^{-1}{\text{cm}}^{-1}).$ The MRW was calculated with 110.2 for PiL[D24] (688 aa, MW: 75.8 kDa) and 108.9 for PKM2 (551 aa, MW: 60.0 kDa).

### Fluorescence emission scans of PiL[D24] measured by fluorometry

Fluorescence emission spectra of purified His‐PiL[D24] were collected on a fluorometer at an excitation wavelength of 450 nm using 100 μL of 0.4 mg·mL^−1^ protein in a quartz cuvette with 1 cm optical path length. The temperature during the experiment was set at 4 °C to slow down the recovery reaction of the LOV2 photocycle 48, 49. The duration of a full emission scan (~ 15 s acquisition time) was sufficient to switch off the fluorescence of the LOV2 domain. The fluorescence recovery kinetics for LOV2 in PiL[D24] were followed by increasing the time delay before the second emission scan (from 15 s to 10 min as indicated in Fig. 4D) to allow partial fluorescence recovery. The fluorescence signal at the emission maximum of 488 nm plotted against the recovery time showed first‐order exponential decay kinetics. The emission spectrum of free FMN was recorded in SEC‐buffer under the same conditions to compare with the spectrum of FMN associated with PiL[D24].

### Repeated fluorescence switching of PiL[D24] using a plate reader

Fluorescence of 100 μL 0.4 mg·mL^−1^ purified His_6_‐PiL[D24] was measured at a frequency of 5 Hz (0.2 s per cycle) for 8 s on a fluorescence plate reader with excitation at 450 nm and emission at 495 nm at RT. For repeated cycling between fluorescence decay and recovery, a sequence of excitation intersected by pausing times (without excitation) varying between 20 and 60 s to allow recovery was performed.

### Measurement of pyruvate kinase activity by an LDH‐coupled assay

Steady‐state kinetic measurements of mPKM2 and PiL[D24] in the dark state were performed using a Tecan Infinite 200‐Pro plate reader (Tecan, Männedorf Zürich, Switzerland) equipped with dual‐beam optics and a Peltier system for temperature control. Initial velocities for the forward reaction were measured using a coupled reaction with LDH (supplied in excess so that it is not rate‐limiting), monitoring the reduction of NADH (ε_340_ = 6220 m ^−1^ cm^−1^) at 37 °C in a buffer containing 10 mm Tris/HCl pH 7.4, 100 mm KCl, 5 mm MgCl_2_ and 1 mm DTT. All chemicals and LDH were from Sigma (St. Louis, MO, USA). Initial velocity versus substrate concentration curves for phosphoenolpyruvate and ADP were individually measured in the absence and in the presence of 400 μm FBP, in a reaction buffer containing 180 μm NADH and 8U LDH. The initial velocity versus FBP concentration was measured following a 2‐min incubation in the desired concentration of FBP in reaction buffer. Reactions were initiated by adding the ADP and phosphoenolpyruvate at their desired concentrations. For reactions where phosphoenolpyruvate was varied, ADP was added at a constant concentration of 2.5 mm; and when ADP was varied, phosphoenolpyruvate was added at a constant concentration of 10.0 mm. A total protein concentration of 35 nm for PiL[D24] and 5 nm for mPKM2 was used for all enzyme reactions, in a total reaction volume of 100 μL per well.

### Measurement of pyruvate kinase activity by NMR spectroscopy

Pyruvate kinase activity of PiL[D24] with and without continuous blue light illumination was monitored in real time by one‐dimensional (1D) ^1^H NMR. The sample was exposed to blue light with an optic fibre (FP1000URT (5 m, without tubing, end 1: SMA, end 2: flat cleaved), Thorlabs) coupled to an LED light source (Optogenetics‐LED‐460, Prizmatix). The fibre was positioned with a coaxial insert (WSG‐5BL, Wilmad, Vineland, NJ, USA) with the stem cut‐off and a 2.52 × 1.5 mm insert (Wilmad, 519‐INNER) in the 5 mm NMR tube (528‐PP‐7, Wilmad) submerging 1 cm into the reaction volume. Purified PiL[D24] at a 0.38 mg·mL^−1^ (5 μm) stock was diluted 1 : 100 into 500 μL PK assay buffer (50 mm Tris/HCl pH 7.5, 100 mm KCl, 5 mm MgCl_2_) containing 1 mm DTT, 3 mm ADP, phosphoenolpyruvate at 0.1, 0.2, 0.5, 1, 2 and 3 mm, and 5% D_2_O. Spectra were acquired on a Bruker Avance III spectrometer with a 11.7 T magnet and a 5 mm TCI cryoprobe. Solvent suppression was achieved using excitation sculpting 89. Data were recorded in pseudo‐2D mode using eight scans per ‘increment’ over 30‐s intervals, with no dummy scans. The acquisition time was 3 s and the relaxation delay 0.75 s. To circumvent the settling time of the standard temperature regulation system, the probe temperature was chosen to closely match the room temperature [294.2 K, (21 °C)] and the sample was inserted without use of the pneumatic sample ejection/injection system by lowering in and pulling out the tube together with the optic fibre. Automated locking and shimming of the sample was completed within 1 min after sample insertion. The spectra were processed in Topspin 3.5 using 2 Hz line broadening and localised polynomial baseline correction around the peaks of interest. The Bruker Dynamics Centre software was used to extract the time‐dependent peak intensities of the reaction product pyruvate. To determine the *K*_m_ for phosphoenolpyruvate under Lit and Dark conditions, initial slopes were determined.

### Testing for effects of light on sample temperature

The resonance frequency of water is temperature‐dependent, so we examined the chemical shift of the water peaks to ask whether there is evidence for a temperature difference under Dark and Lit conditions. Figure 8E shows the overlay of two NMR spectra for water in the experiment of Fig. 8A at *t* = 4 min (Dark) and *t* = 12 min (Lit). In the Lit state, the water peak moved 0.00082 p.p.m. upfield, which is indicative of a temperature increase caused by light. We used this measurement to calculate the temperature change between Lit and Dark conditions, based on the following equation by Gottlieb *et al*. 90 that describes the dependence of the water chemical shift on temperature:

(1) $$\delta_{H_{2}O}=5.051-0.0111T,$$

where $\delta_{H_{2}O}$is the chemical shift of water at temperature *T* in °C.

So, for the Dark and Lit conditions, Eqn (1) is respectively:

$$\delta_{H_{2}O}^{\mathrm{Dark}}=5.051-0.0111T^{\mathrm{Dark}}$$

$$\delta_{H_{2}O}^{\mathrm{Lit}}=5.051-0.0111T^{\mathrm{Lit}}$$

and the difference in chemical shift as a function of temperature can be expressed as:

$$\Delta \delta_{H_{2}O}^{\mathrm{Dark}-\mathrm{Lit}}=0.0111\left(T^{\mathrm{Lit}}-T^{\mathrm{Dark}}\right).$$

Solving for ∆*T*_Lit–Dark_ and substituting $\Delta \delta_{H_{2}O}^{\mathrm{Dark}-\mathrm{Lit}}$ = with the observed value (0.00082 p.p.m.):

$$\Delta T^{\mathrm{Lit}-\mathrm{Dark}}=\frac{\Delta \delta_{H_{2}O}^{\mathrm{Dark}-\mathrm{Lit}}}{0.0111}$$

$$\Delta T^{\mathrm{Lit}-\mathrm{Dark}}=\frac{0.00082}{0.0111}=0.074.$$

So, under Lit conditions, the temperature in the reaction increases by 0.074 °C.

To validate this calculation, we then measured the chemical shift of the water peak in the PK reaction buffer (without the enzymes) at 21.1 °C or at 22 °C, i.e. + 0.1 or + 1 °C, respectively, of the temperature we performed all NMR‐based activity assays at (21 °C; Fig. 8F). The water chemical shift was 4.69739, 4.69632 p.p.m. ($\Delta \delta_{H_{2}O}^{21.1-21.0}$ = −0.001 p.p.m.) and 4.68682 p.p.m. ($\Delta \delta_{H_{2}O}^{22.0-21.0}$) = −0.01 p.p.m.) at 21, 21.1 and 22 °C respectively. The upfield shift of 0.001 p.p.m. at 21.1 °C versus 21 °C is comparable to the $\Delta \delta_{H_{2}O}^{\mathrm{Dark}-\mathrm{Lit}}$ of 0.00082 supporting the conclusion that the temperature increase in Lit versus Dark conditions is ~ 0.1 °C.

We then tested whether the PiL[D24] reaction rate is sensitive to this temperature increase. We measured PiL[D24] activity at 21.0 °C for 8 min then increased the temperature in the spectrometer by 1 °C (confirmed by the change in the chemical shift of water, Fig. 8G). As shown in Fig. 8H, we did not observe a difference in the rate of production of pyruvate, indicating that the PiL[D24] reaction rate is not sensitive to temperature increases of up to 1 °C, i.e. 10 times higher than the actual temperature increase we observed with light.

### Cell line culture and transfection

HeLa, A549 and HEK293T cells were cultured in Dulbecco's modified Eagle's medium (DMEM) (11960; Gibco, Waltham, MA, USA) and H1299 in RPMI medium (31840; Gibco), all containing 10% FCS, 2 mm glutamine (25030081; Gibco) and 100 U·mL^−1^ penicillin/streptomycin (15140122; Gibco). All cell lines were tested mycoplasma‐free and cell identify was confirmed by short tandem repeat (STR) profiling by The Francis Crick Institute Cell Services Science Technology Platform. Cells were transfected with FuGENE (E2691; Promega, Madison, WI, USA) according to the manufacturer's protocol using a 3 : 1 ratio of FuGENE to DNA.

### Production of retroviruses in 293T cells and infection of target cells

Retroviruses were produced in 293T cells by cotransfection of a plasmid expressing the amphotropic receptor gene and the pLHCX‐PiL[D24] or pLHCX‐empty vector (pLHCX‐EV). Viral supernatants were harvested 48 h post‐transfection, filtered through 0.45 μm syringe filters (Millipore, Billerica, MA, USA), supplemented with 4 μg·mL^−1^ polybrene and applied to HeLa cells for 6 h before replacing with regular cell culture media. After 16 h recovery, cells were selected with hygromycin (200 μg·mL^−1^) in media for at least 10 days.

### Cell lysis and western blotting

Cells on cell culture dishes were washed twice with PBS, snap‐frozen in liquid nitrogen and stored at −80 °C. Cells were scraped from the cell culture plate with PK lysis buffer (50 mm Tris/HCl pH 7.5, 1 mm EDTA, 150 mm NaCl, 1% Igepal‐630) supplemented freshly prior to use with protease inhibitors [1 mm 4‐(2‐aminoethyl)benzenesulfonyl fluoride, 4 μg·mL^−1^ aprotinin, 4 μg·mL^−1^ leupeptin and 4 μg·mL^−1^ pepstatin (pH 7.4)] and 1 mm DTT and lysed for 20 min on ice. Lysates were centrifuged at 20 000 ***g*** for 10 min at 4 °C, supernatants were boiled in SDS sample buffer for 10 min, resolved using SDS/PAGE and proteins were transferred to poly(vinylidene difluoride) membranes by electroblotting. Membranes were blocked with 5% milk in Tris‐buffered saline (50 mm Tris/HCl pH 7.5, 150 mm NaCl) containing 0.05% Tween 20 (TBS‐T) and incubated with the primary antibody overnight at 4 °C. Membranes were washed with TBS‐T and incubated with the secondary antibody conjugated to horseradish peroxidase for 1 h at RT in 5% milk TBS‐T. Blots were developed using enhanced chemiluminescent substrate. Primary antibodies used were as follows: rabbit anti‐PKM2 antibody (#4053S, Cell Signalling, Danvers, MA, USA), 1 : 2000 in 5% BSA TBS‐T; mouse anti‐α‐tubulin antibody (T9026, Sigma), 1 : 2000 in 5% milk TBS‐T; and rabbit‐anti‐iLOV antibody (gift from John M. Christie 91), 1 : 2000 in 5% milk TBS‐T. Secondary antibodies were as follows: goat anti‐rabbit IgG antibody conjugated to HRP and goat anti‐mouse IgG antibody conjugated to HRP.

### Live‐cell imaging

To visualise PiL[D24] expression, test its functionality and measure the kinetics of the photoreaction in cells, 30 000 cells per well were seeded in eight‐well μ‐slides glass bottom (80826; Ibidi, Munich, Germany) 16–18 h before the experiment. At the time of the experiment, media were replaced with imaging buffer (20 mm HEPES pH 7.4, 115 mm NaCl, 1.3 mm MgCl_2_, 1.2 mm CaCl_2_, 1.2 mm K_2_HPO_4_, 25 mm glucose). Images were acquired with a Leica SP5 inverted confocal microscope with a 20×/0.7 imm objective, 300 μm pinhole, 490–550 nm emission window and HyD detector using the Argon 488 laser at 20% power and the 476 nm laser line at 10% transmission.

From the available built‐in experimental suite in the lasaf software (Leica Microsystems, Wetzlar, Germany), the fluorescence recovery after photobleaching (FRAP) wizard was used to acquire a time course with images of consecutive LOV2 photocycles. By fast frame acquisition (15 s of 0.65 s per frame), near‐continuous illumination was achieved for switching of PiL[D24]. This was followed by a low imaging frequency (3 min of 15 s per frame or 30 s per frame) to allow recovery of LOV2 fluorescence in PiL[D24].

### Image analysis

Images of the time series were read as a stack into the image analysis software imagej Fiji 92. The background was defined from an area of the image without cells and subsequently subtracted from the images. A maximum projection image of the stack was generated before identification of cells to account for cell movement. The particle analyser function was then used to identify single cell areas that were read into the ROI manager to quantify the mean fluorescence value for identified cells in each frame.

### BlueCell LED device

To illuminate large surface areas (cell culture dishes and microtitre plates), we used BlueCell, a device that comprises hardware and software components built in‐house. The hardware consists of an enclosure with an LED array that is assembled out of densely packed flexible high‐intensity blue LED (460 nm, 1820 lm) strips (769‐–3176, JKL Components, RS, Corby, UK) with an LED viewing angle of 120° for maximal light diffusion. To counteract heat produced by the LEDs, the LED array is mounted on a heat sink (903–3078, ABL Components; 163AB2000B, RS, Corby, UK) and has cooling fans. The LED array is operated with a control unit that consists of a Raspberry Pi microcomputer, a custom‐built Analogue to Digital and Digital to Analogue Converter‐Printed Circuit Board (ADDAC‐PCB) and an LED‐driver (2289681; RCD‐48‐1.2/M, Farnell, Leeds, UK) to allow variable light intensity outputs from 1 to 10 mW·cm^−2^. Illumination sequences consisting of multiple intervals of continuous or pulsed light at various light intensities can be generated through a graphical user interface (GUI). The light sequence is stored in an output file that is executed via a Python program stored on the Raspberry Pi to control the state of the LED array. To monitor the actual light intensity to which cells are exposed during the experiment and record possible LED‐heat associated fluctuations of temperature, optical and temperature sensors are embedded close to the position of the cell culture dish and are read with the ADDAC‐PCB. These readings are shown in real time on the terminal together with the designed light sequence and are additionally written into an output file to allow postexperiment inspection. During experimental illumination, the temperature was recorded to be stable at 37 ± 0.2 °C.

### ^13^C‐glucose labelling of cells and GC‐MS

HeLa‐PiL[D24] and HeLa empty vector (HeLa EV) were seeded 16–18 h before the experiment at 600 000 cells per well in six‐well cell culture dishes using custom‐made DMEM (cDMEM) devoid of riboflavin with added 2 mm glutamine (25030081; Gibco), 100 U·mL^−1^ penicillin/streptomycin (15140122; Gibco), 10% dialysed FCS (3.5 kDa MWCO) and 5 mm glucose. For the experiment, media were changed and cells were either illuminated for 1 h at 1 mW·cm^−2^ in the cell culture incubator using the BlueCell^4^ LED unit or kept without light exposure. Then, 5 mm U‐^13^C‐glucose in media were added and cells were quenched at 15, 30, 75, 315, 915 s after two washes with cold PBS by snap‐freezing in liquid N_2_. In parallel, a replicate set of plates without addition of U‐^13^C‐glucose media was sampled. A separate set of plates was seeded with cells that were counted to calculate metabolite concentrations relative to cell numbers. Cells were scraped on ice into 0.75 mL methanol. The material was transferred to a 2 mL Eppendorf tube and residual material in the plate was collected using 0.25 mL water containing 1 nmol *scyllo*‐inositol (I8132; Sigma) and added to the methanol fraction. About 0.25 mL chloroform was added to make a final ratio of chloroform : methanol : water 1 : 3 : 1 (v/v) (all HPLC‐grade; Chloroform: 390760025; Acros Organics, Geel, Belgium; Methanol: A456‐212; Fisher Chemicals, Pittsburgh, PA, USA; Water: W/0106/PB17, Fisher Chemicals), and metabolites were extracted by incubation at 4 °C for 16 h after 3 × 8 min cycles of sonication. Cell debris was pelleted by centrifugation (20 000 ***g***, 10 min, 4 °C) and the supernatant transferred to a fresh 1.5 mL Eppendorf tube. Polar and apolar metabolites were biphasically partitioned by addition of 500 μL H_2_O (final solvent ratio: CHCl_3_ : MeOH : H_2_O 1 : 3 : 3 v/v). The polar (upper) phase was transferred to glass vial inserts, dried in a SpeedVac concentrator and washed twice with MeOH.

Metabolites were analysed as previously described 93, 94. In brief, metabolites were derivatised by methoximation [20 μL of 20 mg·mL^−1^ methoxyamine‐HCl (226904; Sigma) in pyridine (270970; Sigma) at RT, overnight], and subsequent incubation with 20 μL *N*,*O*‐bis(trimetylsilyl)trifluoroacetamide (BSTFA) + 1% trimethylchlorosilane (TMCS; 33148; Sigma) for ≥ 1 h. Metabolite analysis was performed by GC‐MS using an Agilent 7890B‐5977A system (Agilent Technologies, Santa Clara, CA, USA). Splitless injection (injection temperature 270 °C) onto a 30 m + 10 m × 0.25 mm DB‐5MS + DG column (Agilent J&W) was used, with helium as the carrier gas, in electron impact ionisation (EI) mode. The initial oven temperature was 70 °C (2 min), followed by temperature gradients to 295 °C at 12.5 °C/min and then to 320 °C at 25 °C/min (held for 3 min). Metabolites were identified and quantified by comparison to the retention times, mass spectra and responses of known amounts of authentic standards using MassHunter Workstation software (B.06.00 SP01; Agilent Technologies). Label incorporation into individual metabolites was estimated as the percentage of the metabolite pool containing one or more ^13^C atoms after correction for natural abundance isotopes in both the metabolite and the derivatisation reagent. The data were plotted using r software 95. % label incorporation is expressed relative to the initial concentration of U‐^13^C‐glucose.

## Author contributions

SG designed, cloned and purified PiL[D24], and performed all the fluorescence experiments, CD experiments, NMR experiments and GC‐MS. JM did the MD simulations and data analysis. FF and JK supervised computational work. PCD and SRM contributed to the design of and helped with NMR and CD experiments, respectively. JIM provided technical advice and helped with GC‐MS experiments. AS provided technical knowledge, participated in the design of the LED array device, which he also constructed. SG and JM prepared the figures with guidance from DA. DA conceived and supervised the study, designed and interpreted experiments and wrote the manuscript with significant input from SG, JM, FF and JK. All authors reviewed the manuscript.

## Supporting information


**Table S1**. GC‐MS data corresponding to Fig. 10C–D
