# Supplementary material for: An engineered photoswitchable mammalian pyruvate kinase
Source: FEBS J. 2017 Aug 16;284(18):2955–80. doi: 10.1111/febs.14175 (PMC5637921; doi:10.1111/febs.14175)
Supplement: Supplementary file 1 — Table S1. GC‐MS data corresponding to Fig. 10C–D [file FEBS-284-2955-s001.zip › febs14175-sup-0001-TableS1.pdf]

FEBS PRESS
science publishing by scientists
WILEY
The FEBS Journal
www.febsjournal.org

An engineered photoswitchable mammalian pyruvate kinase

Stefanie Gehrig, Jamie A. Macpherson, Paul C. Driscoll, Alastair Symon, Stephen R. Martin, James I. MacRae, Jens Kleinjung, Franca Fraternali and Dimitrios Anastasiou

DOI: 10.1111/febs.14175

Raw data after correction for natural abundance isotopes

NB: Labeling media contained 40:60 [U-^{13}C]-glucose:^{12}C-glucose

Cell line	Condition	Sample name	Pyruvic Acid (174)					PEP (369)				
			Labelling %	M+0	M+1	M+2	M+3	Labelling %	M+0	M+1	M+2	M+3
		Metabolite Mix	0.0084559	0.98642071	0.00614798	0.00307424	0.00435708	0.03154601	0.9142891	0.0763801	0.00973447	-0.00040367
		Metabolite Mix	0.00468597	0.98965232	0.00896568	-0.00094621	0.00232822	0.04883671	0.86604413	0.11615869	0.02304012	-0.00524294
HeLa-EV	DARK	HeLa DARK C12	0.01049553	0.9827412	0.00751835	0.00525314	0.00448731	0.04565644	0.89446982	0.08688299	0.00585522	0.01279197
		HeLa DARK C12	0.01426551	0.96867439	0.02187002	0.00744028	0.00201532	0.03080595	0.92778204	0.05940568	0.00542466	0.00738762
		HeLa DARK C12	0.00672738	0.98863579	0.00493154	0.0040474	0.00238527	0.03874952	0.92529839	0.04770637	0.01244352	0.01455172
		HeLa DARK 5 min C13	0.12542927	0.86406496	0.01213656	0.00724417	0.11655431	0.40705766	0.54301363	0.06702447	0.01573717	0.37422473
		HeLa DARK 5 min C13	0.15732159	0.83312038	0.00918405	0.01030601	0.14738958	0.43254129	0.52003907	0.05342167	0.03541557	0.3911237
		HeLa DARK 5 min C13	0.19814308	0.7901964	0.01071943	0.01354271	0.18554146	0.42664393	0.53216752	0.04892454	0.02571658	0.39319136
	LIT	HeLa LIT C12	0.00714999	0.98650054	0.00824609	0.00255624	0.00269714	0.04242042	0.91615042	0.06014137	0.00400472	0.01970349
		HeLa LIT C12	0.04089762	0.91096507	0.06629751	0.01181689	0.01092053	0.04253636	0.9114289	0.07289389	-0.00768356	0.02336077
		HeLa LIT C12	0.00693782	0.99103917	0.00080277	0.0044635	0.00369456	0.02662193	0.94038834	0.05100998	-0.00305075	0.01165243
		HeLa LIT 5 min C13	0.13485519	0.85100091	0.01602527	0.01038115	0.12259266	0.40782755	0.54397518	0.05864554	0.02730074	0.37007854
		HeLa LIT 5 min C13	0.17517575	0.80331105	0.02545921	0.01362118	0.15760857	0.41200283	0.55038285	0.05002013	0.01280269	0.38679433
		HeLa LIT 5 min C13	0.32028835	0.55947185	0.00920938	0.34230064	0.08901813	0.32881878	0.63850957	0.03865896	0.02069703	0.30213444
HeLa-PiL[D24]	DARK	HeLa D24 DARK C12	0.01218782	0.96424077	0.03714609	-0.00357792	0.00219107	0.0448267	0.89724758	0.08324284	0.00729148	0.01221811
		HeLa D24 DARK C12	NaN	NaN	NaN	NaN	NaN	0.02270332	0.93986225	0.06022481	-0.00814633	0.00805927
		HeLa D24 DARK C12	NaN	NaN	NaN	NaN	NaN	NaN	NaN	NaN	NaN	NaN
		HeLa D24 DARK C12	0.0158987	0.96270665	0.03024982	0.0036843	0.00335923	0.03913348	0.91681343	0.06064429	0.01087072	0.01167157
		HeLa D24 DARK 0 min C13	0.08261926	0.88948884	0.03843155	0.00681262	0.065267	0.27983812	0.68259731	0.05231251	0.00806868	0.25702149
		HeLa D24 DARK 0 min C13	0.10558956	0.86265805	0.04585182	0.00355355	0.08793658	0.26203989	0.68270469	0.06768041	0.03040545	0.21920945
		HeLa D24 DARK 0 min C13	0.1244503	0.83542726	0.05571654	0.00893426	0.09992195	0.30045009	0.65039969	0.06093661	0.02557745	0.26308625
		HeLa D24 DARK 0 min C13	0.01077241	0.98139747	0.01079198	0.00190641	0.00590415	0.06312213	0.89294928	0.05477322	0.02223934	0.03003817
		HeLa D24 DARK 0.25 min C13	0.08598181	0.90476033	0.01143656	0.00490047	0.07890265	0.37306933	0.56797852	0.07311401	0.03062844	0.32827903
		HeLa D24 DARK 0.25 min C13	0.11057603	0.87343514	0.01979576	0.00837496	0.09839414	0.37142845	0.58613188	0.05481832	0.01768239	0.34136741
		HeLa D24 DARK 0.25 min C13	0.10248267	0.87774391	0.02621474	0.00689077	0.08915058	0.32274121	0.6241565	0.06623653	0.02683382	0.28277315
		HeLa D24 DARK 0.25 min C13	0.14034367	0.83819797	0.02288307	0.01860895	0.12031002	0.37648149	0.57809416	0.05116411	0.03394484	0.33679689
		HeLa D24 DARK 1 min C13	0.11186047	0.83034382	0.07366776	0.02605163	0.0699368	0.38462968	0.58255354	0.04108606	0.01627823	0.36008217
		HeLa D24 DARK 1 min C13	0.17661414	0.79586036	0.02977685	0.0230228	0.15134	0.39066858	0.57575455	0.03358994	0.03355074	0.35710477
		HeLa D24 DARK 1 min C13	0.04280003	0.95094891	0.00041905	0.01791508	0.03071696	0.43483407	0.52230074	0.05279093	0.02301373	0.40189461
		HeLa D24 DARK 1 min C13	0.07738218	0.90315109	0.02802472	0.00235074	0.06647345	0.39062707	0.56984704	0.04233741	0.03390286	0.35391269
		HeLa D24 DARK 5 min C13	0.17237869	0.80740064	0.01915885	0.0223443	0.15109621	0.42967018	0.52980206	0.04171686	0.03814959	0.3903315
		HeLa D24 DARK 5 min C13	0.03428729	0.96149801	0.00384732	0.00494943	0.02970523	0.41953279	0.5347399	0.05828141	0.02061913	0.38635957
		HeLa D24 DARK 5 min C13	0.17387836	0.80512382	0.02249319	0.01800708	0.15437592	0.42308521	0.53989487	0.03841085	0.03423807	0.38745621
		HeLa D24 DARK 5 min C13	0.19620401	0.78415368	0.02489002	0.00914688	0.18180942	0.41474023	0.54690204	0.04350448	0.02806422	0.38152926
		HeLa D24 DARK 15 min C13	0.04898657	0.94124129	0.01231628	0.00468388	0.04175855	0.42727939	0.5171367	0.06844249	0.02986677	0.38455405
		HeLa D24 DARK 15 min C13	0.1151438	0.85453303	0.03721576	0.01653801	0.09171321	0.43134325	0.5303418	0.03965711	0.03563063	0.39437046
		HeLa D24 DARK 15 min C13	0.13440031	0.85086556	0.01714729	0.00990784	0.12207931	0.37897344	0.57639822	0.04757308	0.03873886	0.33728984
		HeLa D24 DARK 15 min C13	0.13928659	0.84426549	0.01958836	0.01016702	0.12597912	0.46305553	0.49273513	0.04535384	0.04192034	0.4199907
	LIT	HeLa D24 LIT C12	0.01516167	0.95589911	0.04300778	0.00080211	0.000291	0.06010397	0.88863986	0.07338782	0.00699288	0.03097945
		HeLa D24 LIT C12	0.00725002	0.98334251	0.01334079	0.00154082	0.00177588	0.0574	0.90370422	0.05020904	0.01626926	0.02981749
		HeLa D24 LIT C12	0.01220329	0.96900396	0.02793867	0.0005009	0.00255647	0.04324084	0.88616057	0.10461567	0.00256446	0.00665931
		HeLa D24 LIT C12	0.01158393	0.97099984	0.02533641	0.00157587	0.00208788	0.05085476	0.90069948	0.07234099	0.00065531	0.02630422
		HeLa D24 LIT 0 min C13	0.05459629	0.93626248	0.01062328	0.00617715	0.0469371	0.19923688	0.76341076	0.04958904	0.01287899	0.17412121
		HeLa D24 LIT 0 min C13	NaN	NaN	NaN	NaN	NaN	0.25899622	0.68767478	0.09691099	-0.03383496	0.24924919
		HeLa D24 LIT 0 min C13	0.06806094	0.91443455	0.02304503	0.0064235	0.05609693	0.1848152	0.75969099	0.07611667	0.01424809	0.14994425
		HeLa D24 LIT 0 min C13	0.1021343	0.87469118	0.02939589	0.01073177	0.08518116	0.21743845	0.7391026	0.05960676	0.01116332	0.19012732
		HeLa D24 LIT 0.25 min C13	0.09403023	0.89074089	0.0131774	0.01933186	0.07674986	0.37230275	0.57753781	0.05899134	0.03249564	0.33097521
		HeLa D24 LIT 0.25 min C13	0.10181222	0.89626881	0.00049976	0.00475739	0.09847404	0.36404191	0.599996	0.03723764	0.03341098	0.32935537
		HeLa D24 LIT 0.25 min C13	0.13805843	0.846839	0.01649151	0.01232471	0.12434478	0.37057475	0.59457886	0.03867739	0.02718439	0.33955936
		HeLa D24 LIT 0.25 min C13	0.15915518	0.81990921	0.02009609	0.02261465	0.13738005	0.27026304	0.67659827	0.07352228	0.01237151	0.23750794
		HeLa D24 LIT 1 min C13	0.17480226	0.81203912	0.01099351	0.01748884	0.15947853	0.38412396	0.57505636	0.04782431	0.02681042	0.35030891
		HeLa D24 LIT 1 min C13	0.20370818	0.77683727	0.01958664	0.01919036	0.18438573	0.36782216	0.59332625	0.04650157	0.02355164	0.33662055
		HeLa D24 LIT 1 min C13	0.18731044	0.79815316	0.01455262	0.01450398	0.17279025	0.40018889	0.55643594	0.04877064	0.03258423	0.36220919
		HeLa D24 LIT 1 min C13	0.1673173	0.82469085	0.00508098	0.0138136	0.15641457	0.40584309	0.56192817	0.04102834	0.01462955	0.38241394
		HeLa D24 LIT 5 min C13	0.19149233	0.80234379	0.00061848	0.01725468	0.17978305	0.41005337	0.55573995	0.04218565	0.01824875	0.38382565
		HeLa D24 LIT 5 min C13	0.26087293	0.72183346	0.01412779	0.02362527	0.24041349	0.42182376	0.53745875	0.04179592	0.03856062	0.38218471
		HeLa D24 LIT 5 min C13	0.22012373	0.75891221	0.02346375	0.01596468	0.20165936	0.41671689	0.54752024	0.03765818	0.03197225	0.38284933
		HeLa D24 LIT 5 min C13	0.23018322	0.76001257	0.00298401	0.02344463	0.21355879	0.41694211	0.54199581	0.04828991	0.02660644	0.38310785
		HeLa D24 LIT 15 min C13	0.19835592	0.78432608	0.01882859	0.0142968	0.18254852	0.39790941	0.54855174	0.06798698	0.0246426	0.35881868
		HeLa D24 LIT 15 min C13	0.24990196	0.72883108	0.02225747	0.01928596	0.2296255	0.42083206	0.52643664	0.06223123	0.03373144	0.3776007
		HeLa D24 LIT 15 min C13	0.19562097	0.78980358	0.0121138	0.01949874	0.17858388	0.41606665	0.5563307	0.02688241	0.02904314	0.38774375
		HeLa D24 LIT 15 min C13	0.21987828	0.76033099	0.01954846	0.02027527	0.19984528	0.43656665	0.51182585	0.06041804	0.03398642	0.3937697

Peak areas for metabolite fragment ions and isotopologues quantified in Agilent MassHunter Software

Cell line	Condition	Sample name	Pyruvic Acid (174)				PEP (369)				scyllo-Inositol (318)
			M+0	M+1	M+2	M+3	M+0	M+1	M+2	M+3	M+0
		Metabolite Mix	1721813.06	221853.419	84972.77941	10576.53861	10056130.98	2971445.694	1581727.901	319490.3468	7749120.928
		Metabolite Mix	2499092.59	329069.3497	114020.3732	9828.689967	12983203.24	4493112.126	2388494.381	468207.9695	11699552.48
HeLa-EV	DARK	HeLa DARK C12	379909.4203	49489.4702	19661.6197	3371.517715	61988.52959	19159.35662	9674.542969	2938.060399	13869322.99
		HeLa DARK C12	243742.1643	35389.7496	13629.86742	1792.477916	95066.27218	26235.94644	14103.5172	3483.405932	13828388.25
		HeLa DARK C12	416232.9213	53113.12687	20884.71304	2683.287246	61428.04804	16186.49788	9417.682601	2727.937768	13168680.96
		HeLa DARK 5 min C13	472788.2591	64612.19304	26275.70787	66125.3247	31065.94916	10418.77678	5718.5756	22690.69438	11160498.25
		HeLa DARK 5 min C13	268463.5135	35877.40669	15890.89997	48923.68155	40504.96186	12745.76972	8862.92966	32362.15543	13385109.94
		HeLa DARK 5 min C13	228234.6649	31081.37102	14669.04105	54965.20998	38543.56486	11712.61919	7583.298827	30068.9477	13704550.98
	LIT	HeLa LIT C12	450088.7081	58950.38738	22093.11697	3032.231598	78206.41054	21709.41833	11513.65865	3916.704211	13720481.26
		HeLa LIT C12	53378.13969	10429.73316	3595.844004	1077.849562	77691.98935	22680.09021	10679.34745	4144.122952	13496420.94
		HeLa LIT C12	250578.8335	30927.97909	12547.27521	1909.951783	85141.97627	22663.86432	11680.57581	3225.320384	14379770.5
		HeLa LIT 5 min C13	287124.1795	40612.91154	17221.05714	42986.55521	28536.63742	9124.725794	5763.658913	20661.78823	11438018.05
		HeLa LIT 5 min C13	271313.6657	41866.12732	17991.44012	55083.18092	45559.15042	13796.58565	7811.590106	33650.24599	13444201.97
		HeLa LIT 5 min C13	80618.96979	11212.24118	53153.41856	19202.19691	34281.64073	9341.444375	5971.307264	17382.43374	9261618.842
HeLa-PiL[D24]	DARK	HeLa D24 DARK C12	195955.2544	31576.18662	9108.587288	1347.021522	57468.21379	17511.79036	9006.806804	2671.47247	15114495.89
		HeLa D24 DARK C12	0	0	0	0	62029.31827	17121.57369	8302.648429	2120.418304	14505637
		HeLa D24 DARK C12	0	0	0	0	0	0	0	0	14054418.11
		HeLa D24 DARK C12	273276.6116	42094.91421	14524.59818	2375.449666	50260.53677	13977.07165	7781.006657	2159.020492	14205723.19
		HeLa D24 DARK 0 min C13	208625.7672	34594.83413	12189.33637	16603.41405	33674.96888	9718.023448	5286.982708	13742.93873	12468731.92
		HeLa D24 DARK 0 min C13	138405.111	24327.17699	7765.433041	14970.42252	40957.17606	12741.00988	7965.555166	14846.79195	13287025.34
		HeLa D24 DARK 0 min C13	229025.9664	43356.53492	14735.93525	29144.63038	47887.84742	14636.27763	9008.661256	21266.92937	15343378.96
		HeLa D24 DARK 0 min C13	180975.0222	24180.55304	8824.501367	1820.496466	49543.1136	13539.40123	8265.922176	3270.390854	14653660.1
		HeLa D24 DARK 0.25 min C13	348414.9036	47125.39607	18269.55941	31967.88415	29419.09904	10022.27116	6182.283993	18392.49502	13665413.78
		HeLa D24 DARK 0.25 min C13	311326.1996	45229.58753	18006.33398	36787.32401	31136.38896	9511.267967	5571.144128	19305.93775	13220649.68
		HeLa D24 DARK 0.25 min C13	343199.1029	52331.74201	19556.37457	36788.74601	38013.17835	12090.74578	7390.560243	18817.5739	14638395.52
		HeLa D24 DARK 0.25 min C13	192805.0215	28904.64026	13692.73879	29075.53893	37108.08102	11149.13617	7659.611993	23216.28217	14857987.99
		HeLa D24 DARK 1 min C13	29879.17467	6314.536171	2621.089164	2850.84751	39036.13011	11026.65552	6707.489977	25463.66397	13917420.51
		HeLa D24 DARK 1 min C13	111220.3908	17798.6853	8784.832417	22100.87113	47611.05845	12868.6157	9501.955379	31389.72962	13912835.67
		HeLa D24 DARK 1 min C13	567798.0648	69871.40357	36545.28495	21540.28113	22550.43244	7058.722279	4384.282288	18288.96083	13478231.76
		HeLa D24 DARK 1 min C13	33921.37776	5211.885642	1759.758871	2667.471489	24831.53339	7107.818982	5070.058964	16449.38703	10541247.35
		HeLa D24 DARK 5 min C13	185818.0294	27193.57026	14132.1882	36218.99497	32421.29796	9424.41776	7055.937273	25332.31715	12302497.86
		HeLa D24 DARK 5 min C13	1024098.716	129668.8198	52339.88724	35856.74485	40624.88365	13037.97781	7742.940903	31034.49279	13377836.82
		HeLa D24 DARK 5 min C13	181970.5517	27396.33309	12967.436	36222.99463	38105.2711	10787.24461	7904.249402	28939.02483	13451153.84
		HeLa D24 DARK 5 min C13	124394.1952	19201.15989	7591.356982	29609.9222	50320.65268	14668.10109	9918.774289	37133.40603	13667786.59
		HeLa D24 DARK 15 min C13	2312914.749	313865.4459	120388.8633	113045.2721	25847.75731	8899.240329	5550.591845	20474.29855	13793251.6
		HeLa D24 DARK 15 min C13	274677.6384	45642.38873	19272.29025	31583.51404	22806.08245	6539.007009	4834.197675	17941.10451	12310068.7
		HeLa D24 DARK 15 min C13	881781.1983	125890.9105	52541.33829	131496.3944	31732.89918	9344.724855	6779.362479	19967.56533	13725001.03
		HeLa D24 DARK 15 min C13	378045.7061	55125.78437	22817.82782	58617.54277	28429.15462	8642.196529	6638.838976	25627.45821	13701903.93
	LIT	HeLa D24 LIT C12	362934.4508	60830.74648	18809.38241	2089.85491	67537.92225	19891.94368	10421.73911	4481.940786	13706058.09
		HeLa D24 LIT C12	409006.6659	55699.71082	19918.7934	2421.354445	61078.58916	16338.8096	9694.101622	3857.611727	14078585.44
		HeLa D24 LIT C12	429494.5857	65046.29764	21269.52413	3142.933891	77649.66374	25624.43027	12179.50356	3302.746994	15070139.46
		HeLa D24 LIT C12	459798.6614	68376.30651	23124.3582	3145.390828	64425.34224	18829.08015	9450.368125	3794.546194	14332233.1
		HeLa D24 LIT 0 min C13	129554.7282	17355.48718	6925.854175	7094.732048	34761.96522	9625.68455	5547.12566	9010.940921	13077902.54
		HeLa D24 LIT 0 min C13	0	0	0	0	12287.51062	4335.908209	1346.75336	4784.494635	14002823.28
		HeLa D24 LIT 0 min C13	391686.1396	57898.07032	21771.7331	26109.16117	52730.70155	16459.35066	8907.675295	12310.15582	14626117.02
		HeLa D24 LIT 0 min C13	233400.262	36462.55513	14438.13254	24208.71876	52442.92407	15344.43423	8450.317826	15209.73697	14454228.84
		HeLa D24 LIT 0.25 min C13	276565.3473	38002.80595	19079.42876	25666.09542	27824.93086	8739.487079	5755.625714	17177.97614	13780438.42
		HeLa D24 LIT 0.25 min C13	153207.7918	18871.17278	7790.056823	17443.07781	19904.33208	5453.960212	3936.603194	11702.11401	10547603.24
		HeLa D24 LIT 0.25 min C13	267498.7112	38008.97477	16695.02704	40876.25426	35464.85875	9823.610373	6683.151557	21575.18793	13383343.73
		HeLa D24 LIT 0.25 min C13	359860.5296	52944.93194	27369.99084	63103.98183	39299.34993	12599.77052	6690.82046	15252.19131	13273841.11
		HeLa D24 LIT 1 min C13	312013.0075	42481.90317	21424.9733	63324.33244	37867.51059	11175.08513	7315.452442	24574.56436	14198948.18
		HeLa D24 LIT 1 min C13	247663.7882	36611.99506	18145.01935	60636.75345	54575.09004	15844.23533	10109.60906	33020.51148	14085333.35
		HeLa D24 LIT 1 min C13	190271.7557	26799.5821	12534.63764	42402.04448	37167.40827	11135.12569	7659.174167	25788.29168	14129926.85
		HeLa D24 LIT 1 min C13	284833.3963	36680.04436	17937.51387	55628.98135	38721.37023	11034.00485	6599.914105	27672.42254	14436404.54
		HeLa D24 LIT 5 min C13	235582.2496	29067.77616	15800.46404	54195.55699	38276.77649	11018.14356	6807.994228	27811.45427	12945261.05
		HeLa D24 LIT 5 min C13	254976.5872	36254.66417	20550.94969	87015.27842	53747.31581	15571.20807	11672.04468	40606.80855	15283937.15
		HeLa D24 LIT 5 min C13	229222.002	35193.30795	16113.67507	62580.33598	44951.75121	12619.08393	9076.163214	33249.51621	14195963.12
		HeLa D24 LIT 5 min C13	168356.5352	21304.23652	12929.62916	48530.42639	43081.85401	12969.44796	8483.256245	32221.90331	14356216.68
		HeLa D24 LIT 15 min C13	559829.3234	82083.40398	37307.95044	134010.508	37397.68988	12561.30798	7484.331921	26133.72071	13689665.78
		HeLa D24 LIT 15 min C13	277611.7051	42517.53888	21008.54135	89668.10313	36821.21	12156.8075	8029.469071	28178.65086	12738625.05
		HeLa D24 LIT 15 min C13	201519.0936	27800.33471	14517.17892	46982.45088	30844.14213	8027.696074	5903.046601	22622.4946	12979209.15
		HeLa D24 LIT 15 min C13	224514.2512	33301.42852	16903.42578	60749.9072	36953.89872	12194.40565	8143.090272	30221.68243	13939400.58
